# Chemical reaction network designs for asynchronous logic circuits

**DOI:** 10.1007/s11047-017-9665-7

**Published:** 2017-12-22

**Authors:** Luca Cardelli, Marta Kwiatkowska, Max Whitby

**Affiliations:** 1 Microsoft Research, Cambridge, UK; 2 Department of Computer science, University of Oxford, Oxford, UK

**Keywords:** Chemical reaction networks, Chemical circuits, Chemical reaction network validation, Chemical reaction network simulation, Asynchronous circuit design

## Abstract

Chemical reaction networks (CRNs) are a versatile language for describing the dynamical behaviour of chemical kinetics, capable of modelling a variety of digital and analogue processes. While CRN designs for synchronous sequential logic circuits have been proposed and their implementation in DNA demonstrated, a physical realisation of these devices is difficult because of their reliance on a clock. Asynchronous sequential logic, on the other hand, does not require a clock, and instead relies on handshaking protocols to ensure the temporal ordering of different phases of the computation. This paper provides novel CRN designs for the construction of asynchronous logic, arithmetic and control flow elements based on a bi-molecular reaction motif with catalytic reactions and uniform reaction rates. We model and validate the designs for the deterministic and stochastic semantics using Microsoft’s GEC tool and the probabilistic model checker PRISM, demonstrating their ability to emulate the function of asynchronous components under low molecular count.

## Introduction

Chemical Reaction Networks (CRNs) are traditionally used to capture the behaviour of inorganic and organic chemical reactions in a well-mixed solution. Recently, a paradigm shift in the scientific community has seen the use of CRNs extend to that of a high-level programming language for molecular computing devices (Cook et al. 2009), where the fundamental computational process differs from conventional digital electronics in that it involves transformation of input chemicals into output via reaction rules, as opposed to processing discrete signals (voltage bands) interpreted as Boolean values. Several digital and analogue circuits (Magnasco 1997; Soloveichik et al. 2008) have been designed in CRNs and their computational power studied (Soloveichik et al. 2010; Chen et al. 2013). It has also been demonstrated in principle that any CRN can be physically realised in DNA (Soloveichik et al. 2010; Cardelli 2010; Chen et al. 2013). CRNs are therefore particularly attractive as a programming language for use in nanotechnology and biomedical applications, where it is difficult to integrate traditional electronics.

Chemical systems can store and process information in several ways. We focus on finite systems of molecules interacting in a well-mixed solution under mass-action kinetics and emulate Boolean circuits by encoding information through molecular concentrations reaching a particular threshold. The computation proceeds by transforming input species concentrations into outputs according to the reactions of a finite CRN. It is known that the computational power of CRNs is affected by the choice of the semantics, deterministic or stochastic. In particular, assuming a small probability of error, (finite) stochastic CRNs have been shown to be Turing universal (Soloveichik et al. 2008). The deterministic semantics interprets the reactions as a system of differential equations, which describe the evolution of the system as a vector of real-valued species concentrations over time (Chen et al. 2013). The stochastic semantics, on the other hand, views the state of the system as a vector of (non-negative) integer molecular counts and state transitions as a reaction which has a non-zero probability of occurring (Cook et al. 2009). The stochastic evolution of the system over time is obtained as a solution of the Chemical Master Equation (CME) (Kampen 1992). It is well known that the deterministic semantics is not accurate for small populations. While the stochastic semantics is exact, it is infeasible for large molecular counts. One scalable alternative is the Linear Noise Approximation, which is a real-valued approximation of the CME (Cardelli et al. 2015). The correctness of the behaviour of a circuit described by a finite CRN can be analysed by inspecting its stochastic and deterministic evolution over time. In addition, techniques such as model checking can be employed to analyse the temporal ordering of events.

While CRN designs for synchronous sequential logic circuits have been proposed, to mention (Magnasco 1997; Soloveichik et al. 2010, 2008), a physical realisation of these devices is challenging because of their reliance on a clock to synchronise events in order to ensure the correct temporal order of the phases of the computation. Clocks are difficult to make, since they arise from unique conditions of chemical concentrations and kinetic constants, and must control a large number of events. In electronics, an alternative circuit design technology is asynchronous sequential logic (Spars and Furber 2002; Myers 2004), which instead of a clock relies on handshaking protocols to synchronise events. Asynchronous circuits are widely used for low-power microprocessor designs, e.g., by ARM, though require a larger circuit area. The key component is the Muller C-element, which is used to synchronise multiple independent processes in a manner insensitive to the delays on wires and individual components. To ensure Turing completeness of asynchronous circuits, we also require an isochronous fork in addition to the Muller C-element. An isochronous fork is a component which produces a fan-out of signals that reach the target at virtually the same time. This assumption is difficult to achieve in conventional electronics, because of the need to make the wires the same length, but is straightforward in chemical kinetics because of the well-mixed assumption.

This paper provides novel CRN designs for the construction of an asynchronous computing device based on a bi-molecular reaction motif inspired by the Approximate Majority network (Angluin et al. 2008; Cardelli and Csikász-Nagy 2012). The motif employs catalytic reactions to achieve bistable switching of molecular concentrations, which emulates high and low voltage signals in digital electronics. All components are produced with simple reactions and uniform reaction rates, where we assume a well-mixed solution under mass action kinetics, and are independent of a universal clock. Moreover, any design provided in this paper could in principle be realised as a DNA strand displacement device (Cardelli 2010). We work with the dual-rail design methodology and employ a variant of the diagrammatic language of Cardelli (2014) to represent the designs at the high level. Starting from the Muller C-element, we design the main components of a complete asynchronous computing device in terms of CRNs in a principled way, including logic gates, control flow and basic arithmetic, as well as more complex structures such as queues. We validate the designs by exploring their time evolution for all possible combinations of inputs using Microsoft’s Visual GEC tool,^1^ with the latter also approximated using an experimental implementation of the Linear Noise Approximation (LNA) of Cardelli et al. (2015) provided by Visual GEC that offers better scalability. We use the LNA to highlight a flaw with a key design component. Further, we demonstrate the correct behaviour of the circuits against temporal logic specifications with the probabilistic model checker PRISM^2^ (Kwiatkowska et al. 2011). Our designs constitute the first feasible implementation of asynchronous computational components as CRNs, and are relevant for a multitude of applications in synthetic biology and biosensing.

This paper is an extended version of the conference paper Cardelli et al. (2016).

## Related work

The computational power of Chemical Reaction Networks, viewed as a programming language for engineering biochemical systems, has been studied by a number of authors, to mention Cook et al. (2009) and Chen et al. (2013). There are a number of ways in which chemical systems can encode and process information. This includes simulating Boolean circuits, where information is encoded in binary form using high and low concentrations similarly to this paper, e.g. Magnasco (1997), Soloveichik et al. (2010) and Soloveichik et al. (2008), as well as geometric arrangements, for example self-assembly (Rothemund et al. 2004) and molecular walkers (Dannenberg et al. 2015) not considered here. Researchers have also investigated the power of CRNs to model distributed algorithms (Angluin et al. 2008).

Regarding synchronous logic circuits, much of the work to date considered abstract CRN schemes. One exception is Silva and McClenaghan (2004), where a system of actual chemical reactions is given, together with a precise molecular implementation for gates complete with a thermodynamic analysis of how the system would evolve, though only for simple gate designs. In Cook et al. (2009) we see the construction and composition of simple logic gates based upon catalytic reactions, but they do not mention control flow or systematic component design in a dual rail setting. In Senum and Riedel (2011) the authors propose CRNs for an inverter, an incrementer, a decrementer and a copier; their designs are based on two rate constants, “fast” and “slow”, and thus are not rate-independent, in contrast to the designs presented here.

CRNs can also be viewed as computing functions over reals or Booleans. A single CRN computes a function over a finite domain, which is analogous to Boolean circuits in the sense that any given circuit computes only on inputs of a particular size (Soloveichik et al. 2008). An implementation of dual-rail logic gates that are rate-independent is given in Chen et al. (2014). In contrast, our designs are composable and capable of performing non-trivial computation.

Since the behaviour of CRNs is asynchronous, a fact evident through their equivalence with Petri net models (Cook et al. 2009), the main difficulty with programming them is the need to control the order of reactions. In Cook et al. (2009) it is suggested that this “uncontrollability” can be handled by changing rate constants, an idea followed up in Napp and Adams (2013), where CRN designs for basic arithmetic are given based on two rate constants, “fast” and “slow”. Our designs, on the other hand, exploit the asynchrony of the underlying CRN model and work with uniform rates.

Designs for the Muller C-element, though not the remaining components of an asynchronous device, have been constructed from genetic logic gates (Nguyen et al. 2010) and a genetic toggle switch (Nguyen et al. 2007), but we are not aware of any other nanoscale designs for asynchronous circuits. Soloveichik et al. (2010) shows that any CRN, including those presented in this paper, can theoretically be implemented as a DNA Strand Displacement device. These devices have been demonstrated in the lab (Qian and Winfree 2011, 2011; Chen et al. 2013), and thus provide an indication of experimental feasibility of our designs.

## Preliminaries

### Chemical reaction networks

A *Chemical Reaction Network* (CRN) $C=(\varLambda ,R)$ is a pair of finite sets, where $\varLambda$ is a set of chemical species and and *R* is a set of reactions. $|\varLambda |$ denotes the size of the set of species. Reactions in *R* describe how species interact. Formally, a *reaction* $\tau \in R$ is a triple $\tau =(r_{\tau },p_{\tau },k_{\tau })$, where $r_{\tau } \in {\mathbb {N}}^{|\varLambda |}$ is the vector of molecular counts of the reactants, $p_{\tau } \in {\mathbb {N}}^{|\varLambda |}$ is the vector of molecular counts of the products and $k_{\tau } \in {\mathbb {R}}_{>0}$ are the coefficient associated to the rate of the reaction. We assume ordering of species within vectors is alphabetical. Given a reaction $\tau _1=( [1,1,0]^T,[0,0,2]^T,k_1 )$, where $\cdot ^T$ is the transpose of a vector, we often refer to it as $\tau _1 : A + B \, \overset{k_1}{\rightarrow } \, 2C$, where *A*, *B* and *C* are generic species.

In this paper we are only concerned with uni-molecular reactions, i.e. those which have only one reactant, and bi-molecular, i.e. those with two reactants. The “reversible reaction” notation $A + B \leftrightharpoons 2C$ is a shorthand for the two reactions $A + B \overset{k_1}{\rightarrow } 2C$ and $2C \overset{k_2}{\rightarrow } A + B$, where $k_1$ and $k_2$ are not necessarily equal.

We assume that the system is *well stirred*, that is, the probability of the next reaction occurring between two molecules is independent of the location of those molecules, at fixed volume *V* and temperature; under these assumptions a *configuration* or *state* $x \in {\mathbb {N}}^{|\varLambda |}$ of the CRN is given by the number of molecules of each species. Given a configuration *x* we define $z=\frac{x}{N}$, where $N=V \cdot N_A$ is the volumetric factor, *V* is the volume and $N_A$ Avogadro’s number. We write $x(\lambda _i)$ for the number of molecules of $\lambda _i$ in the configuration *x* and $z(\lambda _i) = \frac{x(\lambda _i)}{N}$ to denote the concentration of $\lambda _i$ in the same configuration.

We will sometimes distinguish between CRNs with different initial configurations, and to this end define a *chemical reaction system* (CRS) as a tuple $S=(\varLambda ,R,x_0)$ where $(\varLambda ,R)$ is a CRN and $x_0 \in {\mathbb {N}}^{|\varLambda |}$ represents its initial configuration, and we sometimes use the terms CRN and CRS interchangeably.

**Diagrammatic CRN notation** To better visualize a CRN $C=(\varLambda ,R)$ as a circuit, we employ a directed multi-edge graph $(\varLambda ,E)$ based upon a fragment of the diagrammatic notation for influence graphs Cardelli (2014). $\varLambda$, the nodes, represent the species of the CRN *C* and edges *E* are derived from reactions *R* as follows. A reaction is represented as a directed multi-edge with sets of species as source and target. Each edge is either a pointed arrow ($\uparrow$) or a rounded arrow (
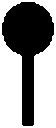
), with the source represented by the flat edge and the target represented by the arrow head. A reaction that produces a species as a product is connected to it by a directed edge.

All reactions within our diagrams are catalytic and are bi-molecular reactions of the form $X + Y \rightarrow X + Z$, meaning that *Y* is transformed to *Z* and *X* is a catalyst, that is, *X* influences the transformation of *Y* to *Z*. The edges 
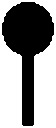
 represent that a source species is catalytic to a target reaction.

#### *Example 1*

**Fig. 1:**
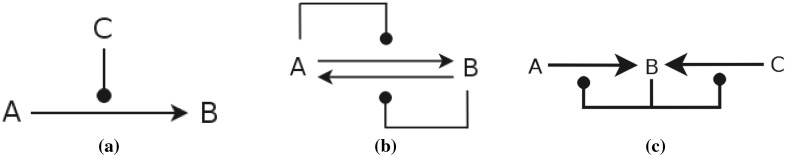
Diagrammatic notation for CRNs. **a** CRN with the single reaction $C + A \rightarrow C + B$, in which species *C* catalyses the reaction $A \rightarrow B$. **b** CRN with reactions $\{ A + A \rightarrow A + B, B + B \rightarrow B + A \}$, in which species A and B are both reactants, products and catalysts. **c** CRN with reactions $\{ A + B \rightarrow B + B, C + B \rightarrow B + B \}$, which demonstrates that B can be catalytic to multiple reactions

(CRN Diagrams Example) We illustrate the flexibility of the diagrammatic notation with three CRN examples. Figure 1a shows the CRN with the single reaction $C + A \rightarrow C + B$. The ball (
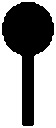
) indicates that C is a catalyst to the reaction $A \rightarrow B$ represented by the arrow ($\uparrow$). A species can act as both a reactant or catalyst in the same reaction, and similarly for a product. In Fig. 1b we depict the CRN with two reactions $\{ A + A \rightarrow A + B, B + B \rightarrow B + A \}$, in which both A and B catalyse themselves. Figure 1c depicts the CRN with reaction set $\{ A + B \rightarrow B + B, C + B \rightarrow B + B \}$, with species B catalysing multiple reactions, which is represented by a multiheaded edge with multiple 
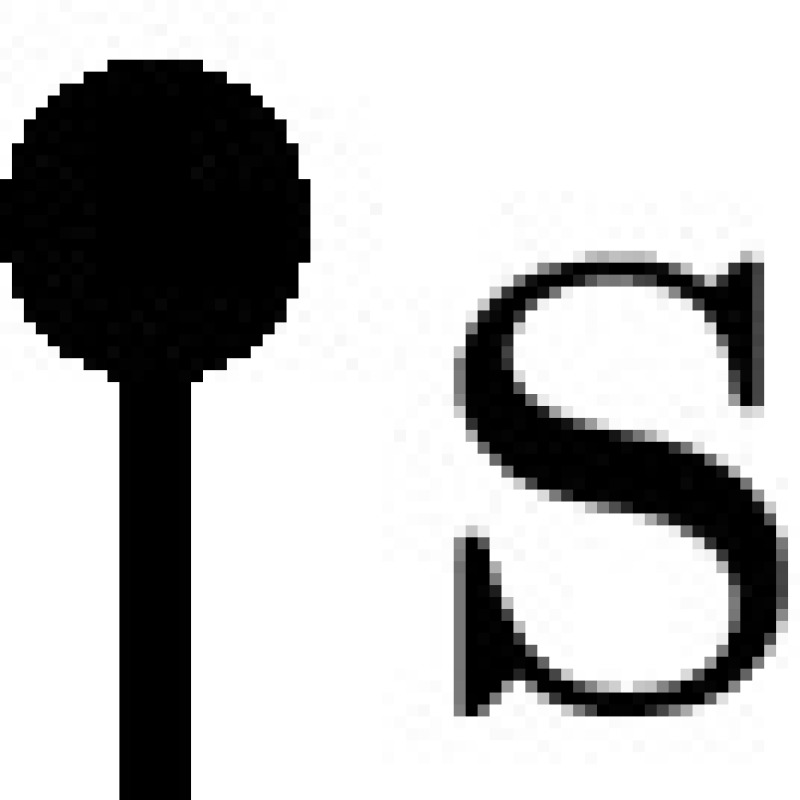
s. This CRN transforms species *A* and *C* into species *B*.

**Dual-rail representation** We represent a Boolean circuit with inputs *I* and outputs *O*, denoted *B*(*I*, *O*), as follows. Firstly, a Boolean variable $b=\{0,1\}$ could be encoded in a single species *X*, where 0 would be encoded as $E[|X|] = 0$ and 1 as $E[|X|] \ge M$, where *E*[|*X*|] denotes an expectation of the number of molecules of *X* and *M* is a molecular population threshold.

The CRN computes by transforming an input concentration into an output concentration, which reaches the appropriate level upon convergence. However, since absence of molecules cannot be measured, we employ *dual-rail methodology* and represent every Boolean variable with two species, denoted $X_{hi}, X_{lo}$. Just like we cannot represent both 0 and 1 on an electrical wire, we restrict our CRNs such that either $E[|X_{hi}|] \ge M$ or $E[|X_{lo}|] \ge M$, but not both, can be present when a CRN has stabilised and no further reactions occur. We consider a high concentration output as correct if $E[|X_{hi}|] > 0.8 max(X_{hi}) - 1SD(X_{hi})$, where 0.8 is a threshold normalised between the values [0, 1], *max*() is a function that returns the maximum molecular concentration of a species within the CRN and 1*SD* computes 1 standard deviation from the mean concentration of $E[X_{hi}]$. 1*SD* returns 0 under the deterministic semantics. Similarly, we consider a low concentration as correct if $E[|X_{lo}|] < 0.2 * max(X_{lo}) + 1SD(X_{lo})$.

For simplicity, we apply the dual rail methodology only to the variables in the input and output sets *I* and *O*. The circuits may contain additional variables, which will be considered internal and assumed not to catalyse with any species outside of the CRN circuit. We will encode these with single species and use the naming convention of referring to these internal species as $\lambda , \lambda _1, \cdots , \lambda _i$. When composing two circuits $B_1(I_1,O_1)$ and $B_2(I_2,O_2)$ in series, we define their composition as a circuit $B(I_1,O_2)$, in which all variables in $O_1 \cup I_2$ have been made internal.

#### *Example 2*

**Fig. 2:**
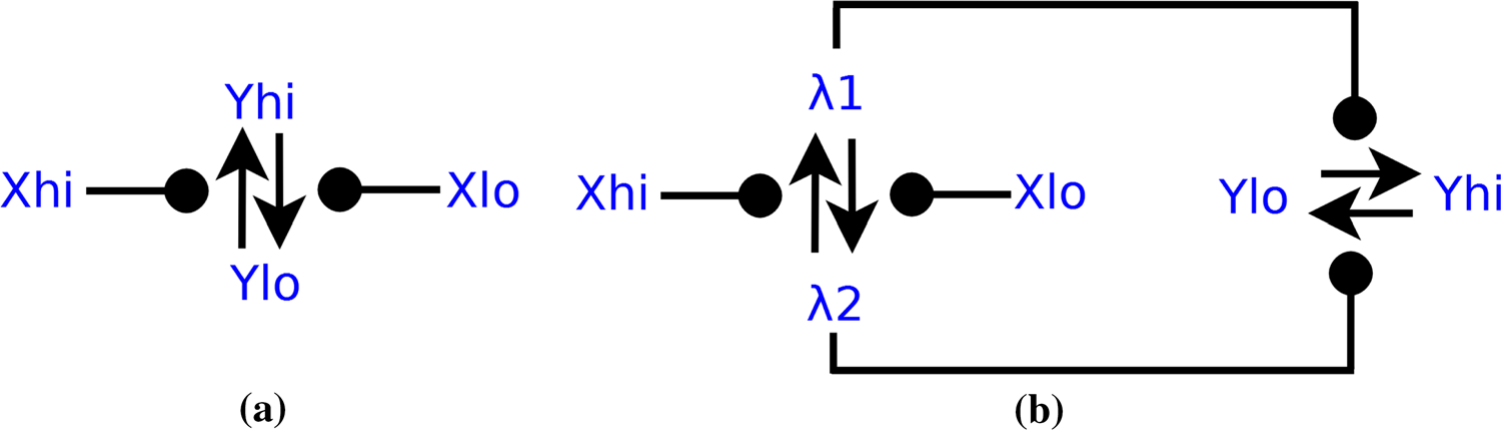
**a** The CRN ‘motif’, in which the input $X_{lo}$ or $X_{hi}$ influences the reaction $Y_{lo} \leftrightharpoons Y_{hi}$ to produce as output the same Boolean value. **b** CRN obtained by composing two ‘motif’s depicted in **a** in series. The inputs are now $X_{hi},X_{lo}$, outputs $Y_{hi}, Y_{lo}$, and the remaining species have been made internal through renaming with $\lambda$.

(Dual-Rail CRN ‘Motif’) We introduce a simple two reaction CRN which forms a ‘motif’ common to all our CRN circuit diagrams. The CRN is given by the set of reactions $\{ X_{hi} + Y_{lo} \rightarrow X_{hi} + Y_{hi}, X_{lo} + Y_{hi} \rightarrow X_{lo} + Y_{lo} \}$ shown in Fig. 2a, where the input set *I* contains $X_{lo}$ and $X_{hi}$ and the output set contains $Y_{lo}, Y_{hi}$. In this CRN the input $X_{lo}$ or $X_{hi}$ influences the reaction $Y_{lo} \leftrightharpoons Y_{hi}$ to produce as output the same Boolean value. We also include a diagrammatic CRN showing the composition of two such motifs in series. Here the inputs are $X_{hi},X_{lo}$ and outputs $Y_{hi}, Y_{lo}$.

#### *Example 3*

**Fig. 3:**
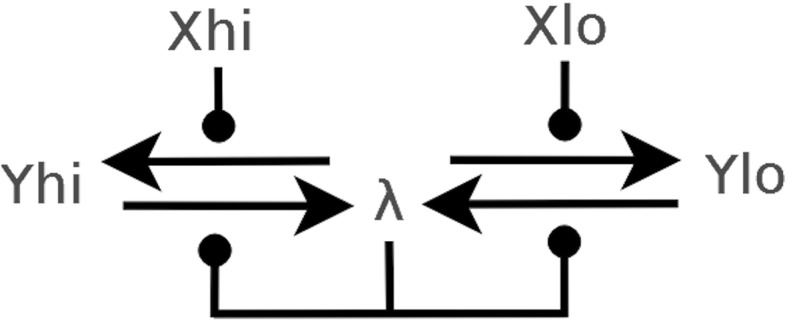
Example CRN with internal $\lambda$ species, which converts outputs $Y_{hi}$ and $Y_{lo}$ to $\lambda$, assuming a non-zero initial concentration of molecules, unless there is input $X_{lo}$ or $X_{hi}$ present

(Dual-rail CRN with Internal ($\lambda$) Species) The CRN shown in Fig. 3 represents a circuit *B*(*I*, *O*) with internal species $\lambda$ which does not belong to the set $I \cup O$. Therefore dual rail methodology is not used for $\lambda$ as it is not catalytic to any species outside of this CRN circuit. *I* comprises $X_{lo}, X_{hi}$ and *O* comprises $Y_{hi}, Y_{lo}$. The CRN is simplified to $\{ X_{hi} + \lambda \rightarrow X_{hi} + Y_{hi}, X_{lo} + \lambda \rightarrow X_{lo} + Y_{lo}, \lambda + Y_{hi} \rightarrow \lambda + \lambda , \lambda + Y_{lo} \rightarrow \lambda + \lambda \}$. This CRN converts $Y_{hi}$ and $Y_{lo}$ to $\lambda$, assuming a non-zero initial concentration of molecules, unless there is either $X_{lo}$ or $X_{hi}$ present, in which case a deadlock occurs. We use the term conversion to mean the occurrence of a reaction where there are non-zero molecular counts of reactants present.

**Deterministic semantics** Let $C=(\varLambda ,R)$ be a CRN. The net change associated to a reaction $\tau \in R$ is defined by $\upsilon _{\tau }=p_{\tau } - r_{\tau }$. The deterministic semantics models the concentration of the species in $\varLambda$ over time as a set of autonomous polynomial first order differential equations (ODEs):

1 $$\begin{aligned} \frac{{\rm d} \varPhi (t)}{{\rm d}t}= & {} \nonumber \\ F(\varPhi (t))= & {} \sum _{\tau =(r_{\tau },p_{\tau },k_{\tau }) \in R} \upsilon _{r} \cdot \left( k_{\tau }\prod _{i=1}^{|\varLambda |}{\varPhi _i(t)}^{r_{\tau ,i}}\right) . \end{aligned}$$

where function $\varPhi : {\mathbb {R}}_{\ge 0} \rightarrow {\mathbb {R}}^{\varLambda }$ describes the behaviour of the system assuming a continuous state-space semantics, and therefore $\varPhi (t) \in {\mathbb {R}}^{|\varLambda |}$ is the vector of the species concentrations at time *t* and *F* is simply the derivative of $\varPhi$ with respect to time. Assuming $t_0=0$, the initial condition is $\varPhi (0)=[x_0]$, where $x_0$ is the initial configuration (vector of concentrations of molecules) of the CRN. It is well known that the deterministic semantics may be imprecise for low molecular counts, but is accurate in the limit for high populations Kampen (1992). However, the deterministic semantics produces the same proportion of molecules, regardless of total concentration.

**Stochastic semantics** The stochastic semantics is represented through a continuous-time Markov chain (CTMC), whose transient evolution can be given via the Chemical Master Equation (CME) (Kampen 1992). Let $C=(\varLambda ,R)$ be a CRN. The propensity rate $\alpha _{\tau }$ of a reaction $\tau$ is a function of the current configuration of the system *x* such that $\alpha _{\tau }(x)dt$ is the probability that a reaction event occurs in the next infinitesimal interval *dt*. We assume mass action kinetics, therefore $$\alpha _{\tau }(x)=k_{\tau } \frac{\prod _{i=1}^{|\varLambda |} r_{i,\tau } ! }{N^{|r_{\tau }|-1}}\prod _{i=1}^{|\varLambda |} \left( {\begin{array}{c}x(\lambda _i)\\ r_{i,\tau }\end{array}}\right),$$ where $r_{i,\tau }$ is the i-th component of the vector $r_{\tau }$ and $|r_{\tau }|=\sum _{i=1}^{|\varLambda |}r_{i,\tau }$Anderson and Kurtz (2011). We define a time-homogeneous CTMC $(X^C(t),t \in {\mathbb {R}}_{\ge 0})$ with state space $Q \subseteq {{\mathbb {N}}}^{|\varLambda |}$ as follows. Given $x_0 \in Q$, where $x_0$ is the initial configuration of the system, then $P(X^C(0)=x_0)=1$. The transition rate from state $x_i$ to state $x_j$ is defined as $r(x_i,x_j) =\sum _{\{\tau \in R | x_j=x_i+ v_{\tau }\}} N\alpha _{\tau }(x_i)$. $X^C(t)$ describes the stochastic evolution of the molecular populations of each species in *C* at time *t*. For $x \in Q$, we define $P^{(t)}(x)=P(X(t)=x|X(0)=x_0)$, where $x_0$ is the initial configuration. The CME describes the time evolution of *X* as:

2 $$\begin{aligned} \frac{\rm d}{{\rm d}t} \left( P^{(t)}(x)\right)= & {} \sum _{\tau \in R} \left\{ N\alpha _{\tau }(x-\upsilon _{\tau })P^{(t)}(x-\upsilon _{\tau })-N\alpha _{\tau }(x)P^{(t)}(x)\right\} . \end{aligned}$$

The solution of the CME is computed through numerical simulation or discretisation techniques such as uniformization (Kwiatkowska et al. 2007), and is generally feasible only for small populations. The CTMC is often represented as a $Q \times Q$ rates matrix, which can be viewed as a state transition graph and subjected to model checking against temporal logic properties (Kwiatkowska et al. 2011).

**Linear noise approximation** The LNA approximates the CTMC as a continuous-state Gaussian process, given in the form of a set of ODEs that describe the time evolution of expectation and variance of the species. The error of approximation is dependent upon the volumetric factor *N*, the structure and the rates of the CRN. Given a CRN ${\mathcal {C}}=(\varLambda ,{\mathcal {R}})$ with initial configuration $x_0 \in {\mathbb {N}}^{|\varLambda |}$ and in a system of volume size *N*, we define the stochastic process $Y=N\cdot \varPhi + \sqrt{N}\cdot Z$, where $\varPhi$ is the deterministic process given in Eq. 1, and *Z* is a zero-mean Gaussian process (since we assume the initial condition is a fixed value), and with covariance *C*[*Z*(*t*)] described by the solution of the following ODEs with initial condition $C[Z(0)]=0$:

3 $$\begin{aligned} \frac{{\rm d} C[Z(t)] }{{\rm d} t}= & {} F'(\varPhi (t), C[Z(t)]) \nonumber \\= & {} J_F(\varPhi (t))C[Z(t)] + C[Z(t)]J^T_F(\varPhi (t))+W(\varPhi (t)) \end{aligned}$$

where ${J}_F(\varPhi (t))$ is the Jacobian of $F(\varPhi (t))$, $J^T_F(\varPhi (t))$ its transpose, and

$$\begin{aligned} W(\varPhi (t))= \sum _{\tau \in {\mathcal {R}}} \upsilon _{\tau } {\upsilon _{\tau }}^T k_\tau \prod _{S \in \varLambda }\varPhi _{S}^{r_{S,\tau }}(t). \end{aligned}$$

Expected value and covariance matrix of *Y*(*t*) are completely characterized by $\varPhi (t)$ and *C*[*Z*(*t*)] since $E[Y(t)]=N\varPhi (t)$ and $C[Y(t)]=\sqrt{N} C[Z(t)] \sqrt{N}=N C[Z(t)]$.

The LNA requires solving a number of ODEs quadratic in the number of species (Cardelli et al. 2015) and is therefore a scalable alternative to the solution of the CME. In contrast to the deterministic semantics, which considers average concentrations, the LNA does not compromise stochasticity.

**Tool support** A number of software tools exist for examining the behaviour of CRNs. We employ Microsoft’s Visual GEC, which provides a programming language, LBS, for designing and simulating a given CRN under the deterministic or stochastic semantics, including also the LNA approximation of the stochastic semantics. The tool is capable of producing plots of expected or average species concentrations over time. This functionality is used extensively within this paper to validate our circuit designs. In addition, Visual GEC exports models to the probabilistic model checker PRISM (Kwiatkowska et al. 2011), which then enables verification of the induced continuous-time Markov chain against temporal logic properties. We use PRISM to verify the correctness of the temporal ordering of events occurring as the CRN circuit executes.

#### *Example 4*

**Fig. 4:**
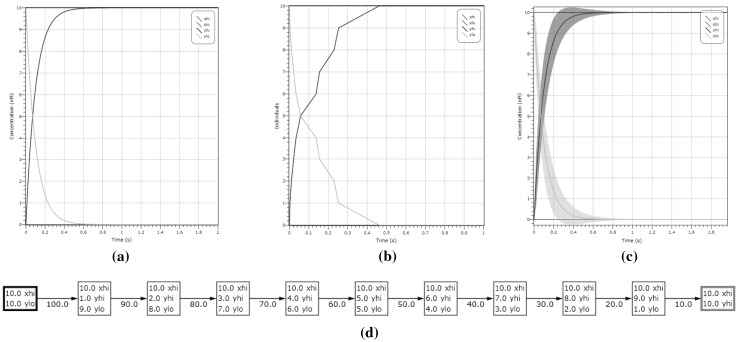
Simulation of the motif given in Example 2 under different semantics. The input is initially $X_{hi}$ and the output is $Y_{lo}$, both starting at a concentration of 10 molecules. In **a**, **b**, **c** we respectively show the deterministic solution, stochastic simulation and LNA plot with variance, where $Y_{lo}$ is seen in blue and $Y_{hi}$ is seen in yellow. We can observe that in all cases $Y_{hi}$ is present after 0.4 s. In **d** we show the state transitions of the induced CTMC that correspond to the output switching from $Y_{lo}$ to $Y_{hi}$. The computation reaches the correct output state and stabilises, with no more transitions enabled. (Color figure online)

(CRN Validation Under Different Semantics) We show the operation of our dual-rail CRN ‘motif’ given in Example 2 under the deterministic and stochastic semantics. The input configuration is $|X_{hi}| = 10$ molecules and output $|Y_{lo}| = 10$ molecules. Figure 4 demonstrates that, after 0.4 s, the CRN stabilises reaching the concentrations of $|Y_{hi}| = 10$ and $|Y_{lo}| = 0$ as desired. With regards to simulations provided, concentrations (given in nanomolars) are directly correlated to concentrations as we assume the volumetric factor *N* is fixed.

### Asynchronous hardware

Asynchronous computation is a model of computation that relies on transitions via local input signals rather than transitions via a global clock. Asynchronous computation (Spars and Furber 2002), just like its synchronous counterpart, is Turing complete (Manohar and Martin 1996), meaning that any bounded-tape Turing machine can be implemented with an asynchronous circuit, providing that the implementation of that circuit has isochronous forks. An isochronous fork is the propagation of a signal from a single source to multiple receivers, with the important constraint that the signal must reach the receivers at precisely the same time. In classical digital circuitry this corresponds to the propagation of a signal down wires of exactly the same length from one component to another. In CRNs this could be seen as two species reaching a threshold *N* at precisely the same time.

Asynchronous circuits, which are designed based upon the theoretical principles of asynchronous computation, are widely used for low-power microprocessor designs, e.g., by ARM, and are increasing in popularity with the increase in distributed computing (Myers 2004). Asynchronous designs offer a number of advantages, the main one being correctness independent of timing, although they require a greater overhead in terms of silicon area.

We illustrate the principles of asynchronous circuit design by describing its key component called the Muller C-element and showing how it is used to construct a pipeline that propagates signals.

**Fig. 5:**
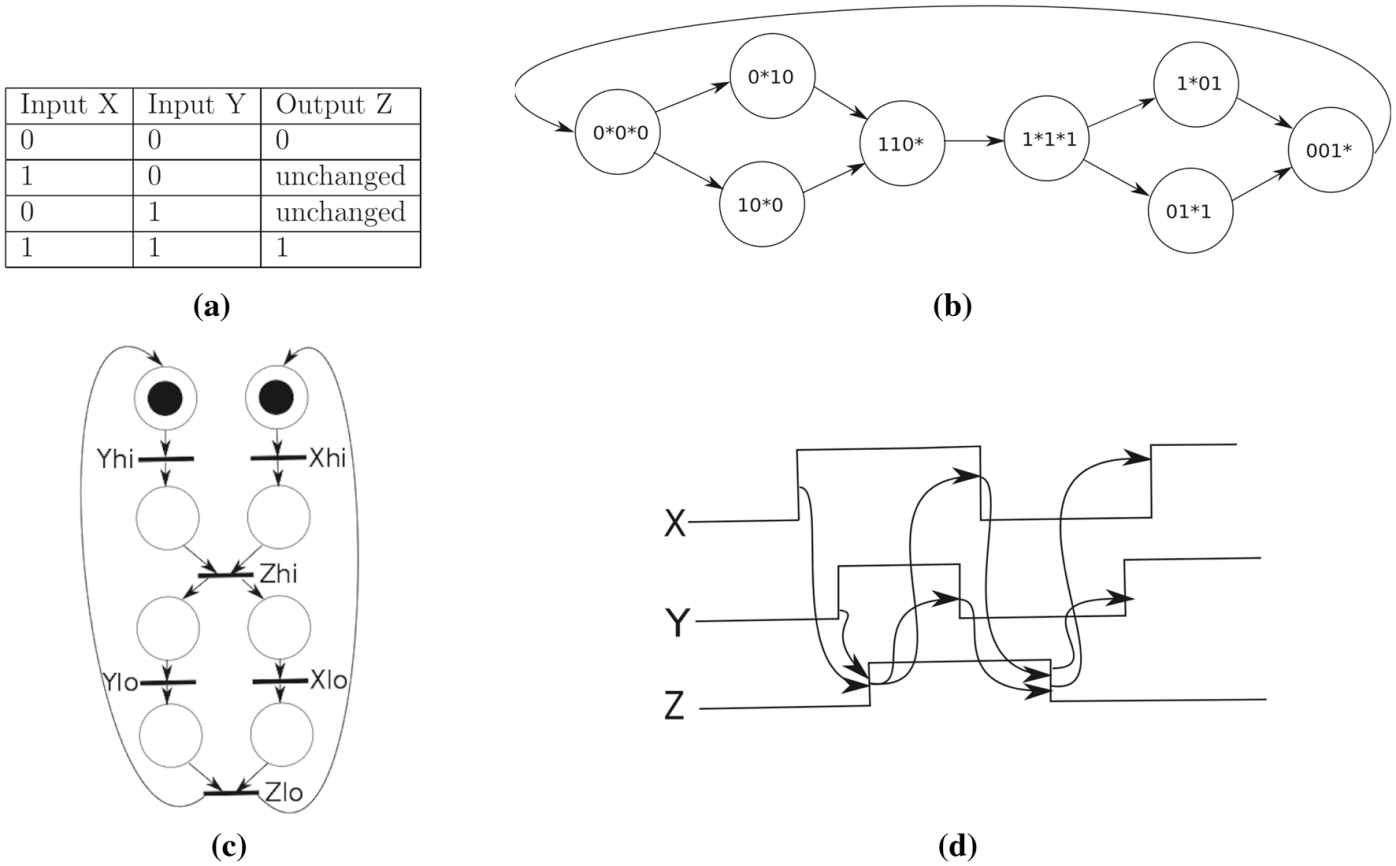
Four specifications of a C-element with inputs X, Y and an output Z. **a** Conventional logic table, where ‘unchanged’ means that the state of the output is the last stable configuration of 1 or 0. **b** State graph, in which each state denotes a configuration and a transition is caused by the presence of a signal, where * indicates that the signal is excited. **c** 1-bounded Petri net specifying the Muller C-element. **d** Timing diagram for the C-element

**Muller C-element** The cornerstone of asynchronous computation is the Muller C-element. A Muller C-element has two Boolean inputs, *X* and *Y*, and one output *Z*. By definition these inputs can either be low or high (represented by 0 or 1). When both inputs are low the output is low. Similarly, when both inputs are high the output is high. The variation from a classical logic gate, however, is that if the inputs are high, or low, and one of them changes, it ‘remembers’ the last state. In other words, it retains the last 0 or 1 state. This is summarised in Fig. 5a. An important property of the C-element is that it allows an observer to conclude on seeing output change from 0 to 1 that *both* inputs are now 1, and similarly for input change from 1 to 0.

The table specification indicates that asynchronous circuits exhibit *concurrency* and *causality*, and hence their specifications need to reflect these characteristics. A common way is as a timing diagram, seen in Fig. 5d for the C-element, which represents a set of signals and their interactions over time. Each row of a timing diagram represents one signal and how it switches from low to high over time. If a signal displays a change before another signal on another line then this signal must precede the other. An arrow represents that one signal change triggers the change of another. In the C-element diagram, note that X and Y have to precede Z, both in the transition to 1 and down to zero. However, there is no causal dependency between X and Y.

Asynchronous diagrams, and in particular the C-element, are accurately described using Petri nets (Spars and Furber 2002, p. 86) or process algebras (Wang and Kwiatkowska 2007). We present a (1-bounded) Petri net for the C-element in Fig. 5c, in which transitions are interpreted as signal transitions and places and arcs capture the causal relations between the signal transitions. Following the usual convention, the Petri net is drawn in simpler form where most places have been omitted. We can observe that both tokens are needed in order to excite the transitions that cause the event $Z_{hi}$, which in turn will require the events $X_{hi}$ and $Y_{hi}$ to be triggered. The same is true for $Z_{lo}$.

When considering circuit synthesis, we typically employ a state graph specification, which can be obtained from the Petri net representation (Myers 2004) and is given for the C-element in Fig. 5b. The values in each state correspond to the values of inputs *X*, *Y* and output *Z*, respectively. A * symbol indicates that the corresponding variable is excited by the outgoing transition (and will be changed in the following state). Observe how we can only transition to a state $1\text {*} 1\text {*} 1$ from a state $110\text {*}$ requiring X as 1 and Y as 1. Because this is derived from a 1-bounded Petri net, we can assume that the transitions $0\text {*}10 \rightarrow 110\text {*}$ and $10\text {*}0 \rightarrow 110\text {*}$ do not conflict.

**Muller C-pipeline** In order to replace the need for a global clock, asynchronous computation relies on ‘local cooperation’ in the form of handshaking protocols. These protocols exchange completion signals in order to establish when a computation has terminated. These handshaking protocols rely heavily on the C-element described above.

**Fig. 6:**
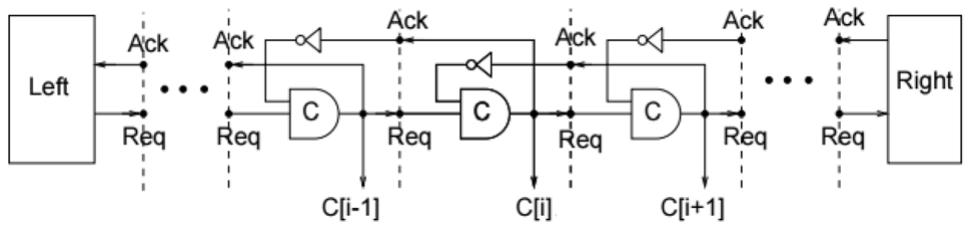
Muller pipeline. Signals are propagated from left to right using request and acknowledge signals. The pipeline effectively queues data, only allowing a transition to occur when a further signal has been acknowledged

The Muller pipeline, shown in Fig. 6, is constructed by the composition of Muller C-elements (depicted by the gate symbol labelled with C) and classical NOT-gates, which receive and send data to/from the environment (Left, Right in the figure). Its function is to propagate a high and low signal along the pipeline, emulating the ‘wave’ of high and low signals of a classical synchronous clock. Initially, all C-elements are set to a value of 0. The *i*th C-element *C*[*i*] will propagate a 1 from its predecessor, $C[i-1]$, only if its successor, $C[i+1]$, is 0. Similarly, it will propagate a 0 from its predecessor only if its successor is 1. Eventually the first request initialised on the left hand side of our pipeline is propagated to the final request on the right.

The protocol enacted upon this pipeline uses request and acknowledge rails that can be set to high or low. The Muller pipeline implements a basic four phase protocol, which is as follows. Firstly, the sender sends data and sets request to high, viewed in Fig. 6 as the signal *Req* being high. The receiver then records this data and sets acknowledge to high (*Ack*). Then the sender responds by setting request to low (*Req*), and finally the receiver acknowledges this by setting acknowledgement to low (*Ack*). If at any point a handshake along the pipeline is slower than another, the pipeline will behave like a FIFO queue with data preserved. Herein lies the important purpose of the pipeline: it allows for the delay-insensitive transfer of information from one place to another. In combination with a latch we can create the propagation of information across latches using the pipeline as a control structure.

The construction of data storage and control structures such as queues and adders is similar to the Muller pipeline.

**Timing properties of asynchronous circuits** Asynchronous circuits can be classified as being self-timed, speed independent or delay-insensitive, depending upon delay assumptions that are made. Assume a circuit is composed of gates and wires. A self-timed circuit operates correctly if both gates and wires experience measurable and fixed delays. A speed independent circuit operates correctly if gates exhibit some unknown time delay within gates but exhibits no time delay on wires. A delay-insensitive circuit operates correctly if there is both unknown delay within gates but also unknown delay within wires. The set of delay insensitive circuits is small, essentially those built from the Muller C-element and NOT gates, and so a broader class of quasi-delay insensitive circuits are identified. Quasi-delay insensitive circuits, which can be composed of purely C-elements, NOT-gates and forks, are Turing complete Manohar and Martin (1996). They are not possible to achieve without an isochronous fork.

## Asynchronous circuit designs as chemical reaction networks

In this section we present our dual-rail designs for an asynchronous computing device in CRNs. The key component is the Muller C-element, whose design is inspired by the well known *Approximate Majority* (AM) CRN (Angluin et al. 2008). We begin by providing a detailed justification for our C-element design, and then describe the remaining simple components, including latches, logic gates and control flow. Finally, we present complex circuits such as the pipeline, queue and adder.

To justify the designs, we demonstrate that each component we design exhibits correct behaviour. Considering as an example the C-element, this amounts to working with an informal specification of the C-element in terms of high/low signals as in Fig. 5, and then showing that our (continuous) CRN empirically satisfies that specification according to appropriate thresholding for high/low signals, as normally done in electronics for transistor logic. To this end, we explore the time evolution of the components under the deterministic and stochastic semantics, including also LNA for scalability. We additionally employ probabilistic model checking with PRISM, where temporal logic is used to express the temporal ordering of events. We remark that, although we validate the components for all possible input configurations, this does not amount to full verification of the correctness of the designs. We discuss the challenges of achieving full verification in Sect. 6.

### Muller C-element as a CRN

**Fig. 7:**
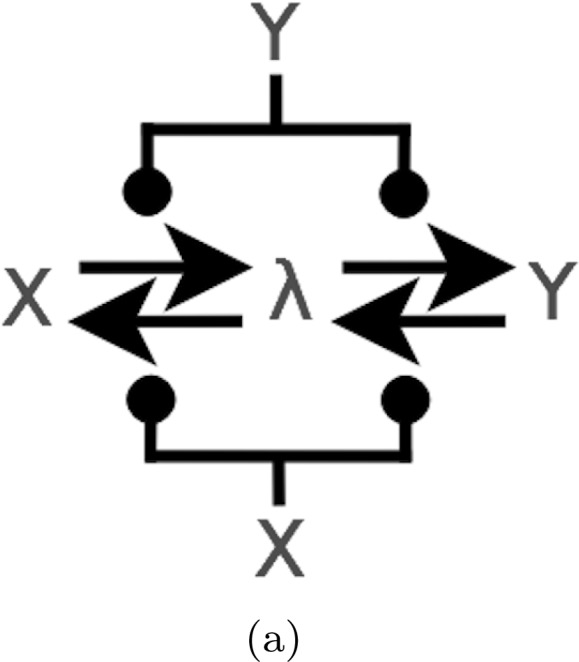
Two diagrammatic CRNs which are capable of computing Approximate Majority Angluin et al. (2008). In **a** we present the original in which the inputs *X*, *Y*, dependent on which species has the majority, influence outputs *X*, *Y*. **b** Shows a similar AM circuit, but now the input species are catalysed to arbitrary output species *W*, *Z*

The C-element design is based on the AM CRN, which computes the majority of two finite populations by converting the minority population into the majority population, so that a single population emerges as output. It uses a third ‘undecided’ state of the population, from where catalysis can drive the individuals into either of the final states. Interestingly, since approximate majority cannot be exactly computed by a bi-molecular CRN with less than 4 reactions (Mertzios et al. 2014), below we present the bi-molecular AM CRN with exactly four reactions:

$$\begin{aligned} X + Y \rightarrow X + \lambda \\ Y + X \rightarrow Y + \lambda \\ X + \lambda \rightarrow X + X \\ Y + \lambda \rightarrow Y + Y \end{aligned}$$

where *X*, *Y* are both the input and output species and $\lambda$ is the aforementioned catalytic driver. The intuition behind this reaction network is that we have two competing initial populations of *X* and *Y*, both of which try to eliminate the other by transforming their counterpart into the intermediary $\lambda$. If $\lambda$ then interacts with *X*, it transforms itself into *X*, else into *Y*. Presented below in Fig. 7a is the same AM CRN in our diagrammatic notation.

Deconstructed, if we consider the left-hand side of the diagram, the reaction $X \rightarrow \lambda$ is catalysed by the species *Y* and so yields the bi-molecular reaction $Y + X \rightarrow Y + \lambda$. Similarly, *X* catalyses the reaction $\lambda \rightarrow X$ in the other direction and yields the reaction $X + \lambda \rightarrow X + X$. On the right-hand side of the diagram, $\lambda$ is again catalysed by *X* and *Y* to produce *Y*, yielding the other two reactions from the AM CRN. The CRN in Fig. 7b, with inputs *X* and *Y* and outputs *W*, *Z*, is similar to the AM CRN, except that we produce new arbitrary outputs *W*, *Z* instead of producing greater quantities of *X* and *Y*.

**Fig. 8:**
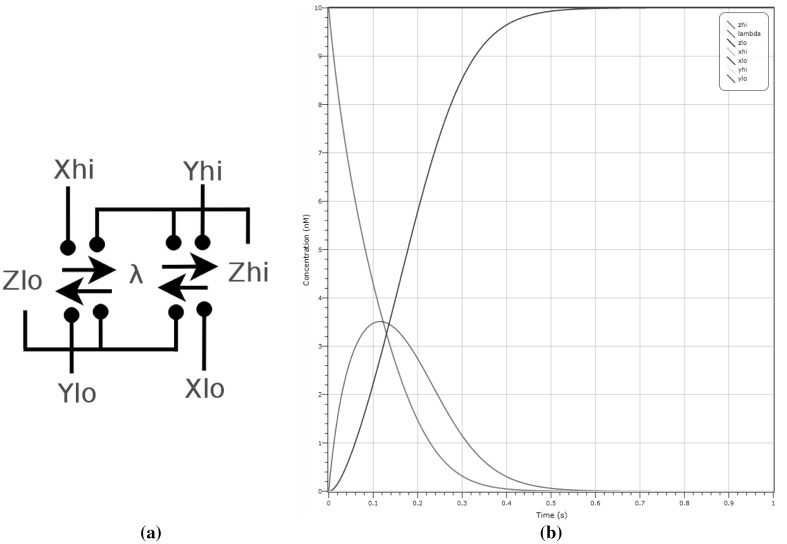
A dual-rail Approximate Majority CRN. **a** Circuit diagram. **b** Deterministic simulation of the CRN in **a**. Given inputs $X_{lo}, Y_{lo}$ at 10 molecules and output $Z_{hi}$, we observe that, because $|X_{lo}|, |Y_{lo}| > |X_{hi}|,|Y_{hi}|$, an output of $Z_{lo}$ where $|Z_{lo}| = 10$ molecules (seen in blue) and $|Z_{hi}| = 0$ (seen in red) is expected to be produced after 0.5 s. (Color figure online)

Since we wish to apply the dual-rail methodology, we represent each signal as a pair of species and encode the value 1 (0) as a molecular population of at least *M* for some population threshold *M* (population 0). We thus present in Fig. 8a a dual-rail CRN which computes approximate majority with four inputs $X_{hi}, X_{lo}, Y_{lo}, Y_{hi}$ and outputs $Z_{hi}, Z_{lo}$. Like before, our input catalyses an intermediary species $\lambda$, but this is split over two reactions. In addition, $Z_{lo}$ and $Z_{hi}$ catalyse with reactions $Z_{lo} \leftrightharpoons \lambda$ and $Z_{hi} \leftrightharpoons \lambda$. This has the effect that, if there are larger numbers of either $Z_{lo}$ or $Z_{hi}$, the network enlarges its majority by converting the other into $\lambda$. Two rounded arrows 
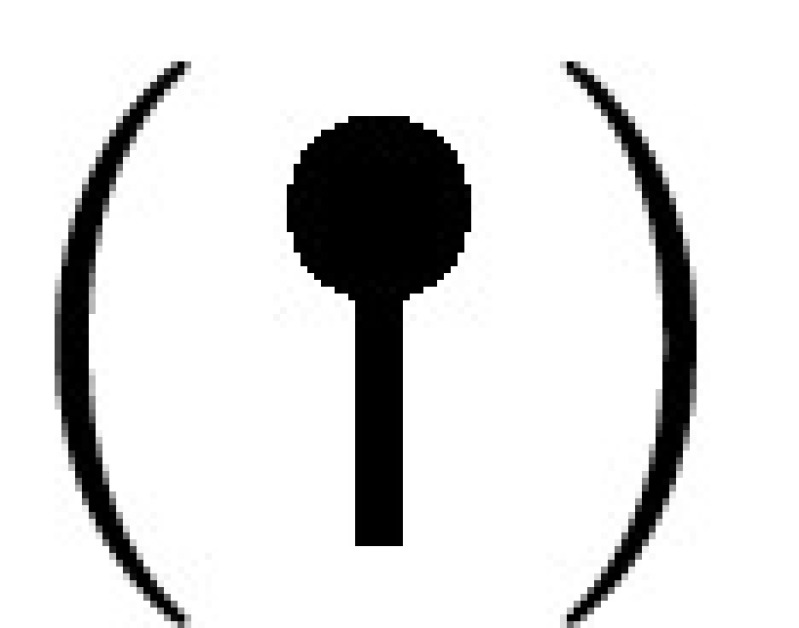
 over a reaction ($\uparrow$) indicates that either species can act as a catalyst to that reaction. We demonstrate this AM effect in Fig. 8b, where, given the initial configuration of inputs $X_{lo}, Y_{lo}$ of 10 molecules and output $Z_{hi}$, we observe under deterministic semantics that, because $|X_{lo}|, |Y_{lo}| > |X_{hi}|,|Y_{hi}|$, an output of $Z_{lo}$ where $|Z_{lo}| = 10$ molecules and $|Z_{hi}| = 0$ is produced after 0.5 s.

We now need to justify the correctness of the design against the C-element specification in Fig. 8a. Given a starting configuration of input *X*, *Y* both 1, and any starting output, the C-element should eventually output 1. Similarly, if *X*, *Y* are both 0, on any initial output our final output should be 0. For any other configuration of *X*, *Y*, the output signal should remain the same. Thus, given an input $X_{hi},Y_{hi}$, and crucially any starting output configuration *Z*, we wish to reach a state where $Z_{hi}$ has at least M molecules where M is a population threshold, and $Z_{lo}$ has 0 molecules. Similarly, for $X_{lo}, Y_{lo}$ and any *Z* we should to see a presence of $Z_{lo}$ after some time *t*. For all other configurations of the inputs we wish the output species to remain the same.

**Fig. 9:**
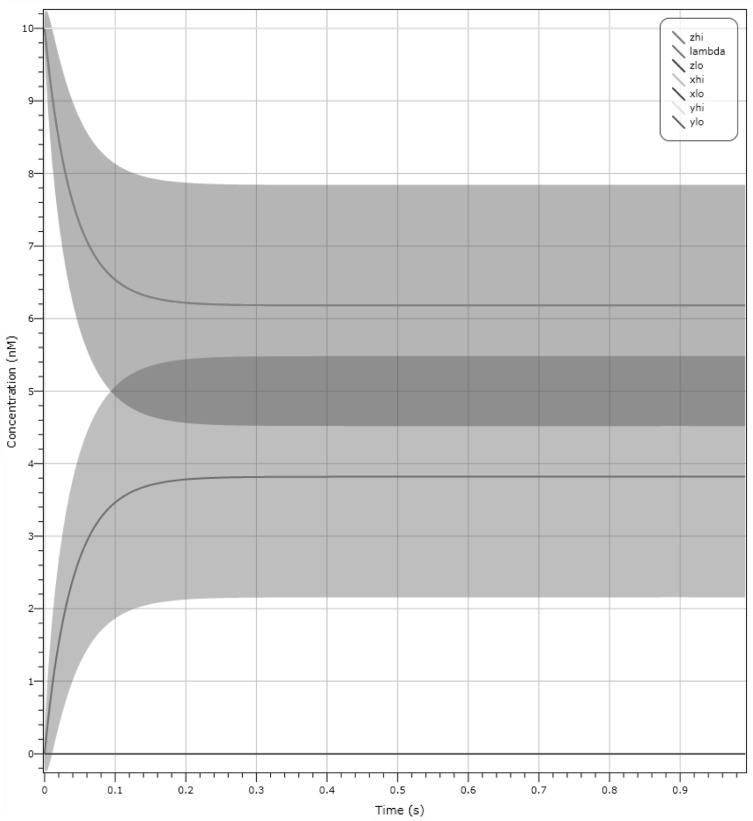
LNA plot for the candidate CRN for the dual-rail Muller C-element design in Fig. 8. With a starting configuration of inputs $X_{lo}, Y_{hi}$ at a concentration of 10 molecules and output $Z_{hi}$ at a concentration of 10 molecules, we can observe that after 0.3 s $Z_{hi}$ (seen in red) is at a concentration of 6 molecules and $\lambda$ is at a concentration of 4 molecules (seen in green). The shaded regions represent standard deviation. As we can see, with a non-zero probability we cannot distinguish the signals. (Color figure online)

When validated against this informal specification with a starting configuration of $X_{lo}, Y_{hi}$ at 10 molecules and $Z_{hi}$ at 10 molecules, our CRN unfortunately fails, seen in Fig. 9. More specifically, we observe that species $Z_{hi}$ is at a concentration of 6 molecules and $\lambda$ has a concentration of 4 molecules after 0.3 s. This simulation was produced using LNA, see Sect. 3.1, which outputs standard deviation of the mean concentrations of species, seen in the shaded regions. This output configuration is incorrect since $Z_{hi}$ is below the required threshold, see Sect. 3.1, of 8 molecules to represent an output of $Z = 1$.

We present an amended CRN that resolves this issue in Fig. 11a. This CRN is composed of two approximate majority circuits connected to each other, with the outcome of the first AM amplifying the outcome of the second. As we can see from the plot in Fig. 9, we need to amplify the $Z_{hi}$ species and suppress the $\lambda$ species. The second AM corrects exactly this issue. Figure 11b is a simulation with inputs $X_{lo}, Y_{hi}$ at 10 molecules and output $Z_{hi}$ at 10 molecules. Here we can see that all three species are now at 10 molecules throughout the duration of the simulation.

To strengthen the validation of the final C-element design, we provide two further plots for selected initial configurations. We include in Fig. 11c a deterministic plot with starting configuration of input $X_{lo}, Y_{lo}$ at 10 molecules and output $Z_{hi}$ at 10 molecules. After 1 s the system converges to output $Z_{lo}$ at 10 molecules. Figure 11d shows an LNA simulation with starting configuration of input $X_{hi}, Y_{hi}$ at 10 molecules and output $Z_{lo}$ at 10 molecules. After 1 s we reach output $Z_{hi}$ with probability $\approx$ 1. Both these simulations show that our output changes if both inputs change. From Fig. 11b we observe that the output does not change if both inputs are different.

**Fig. 10:**
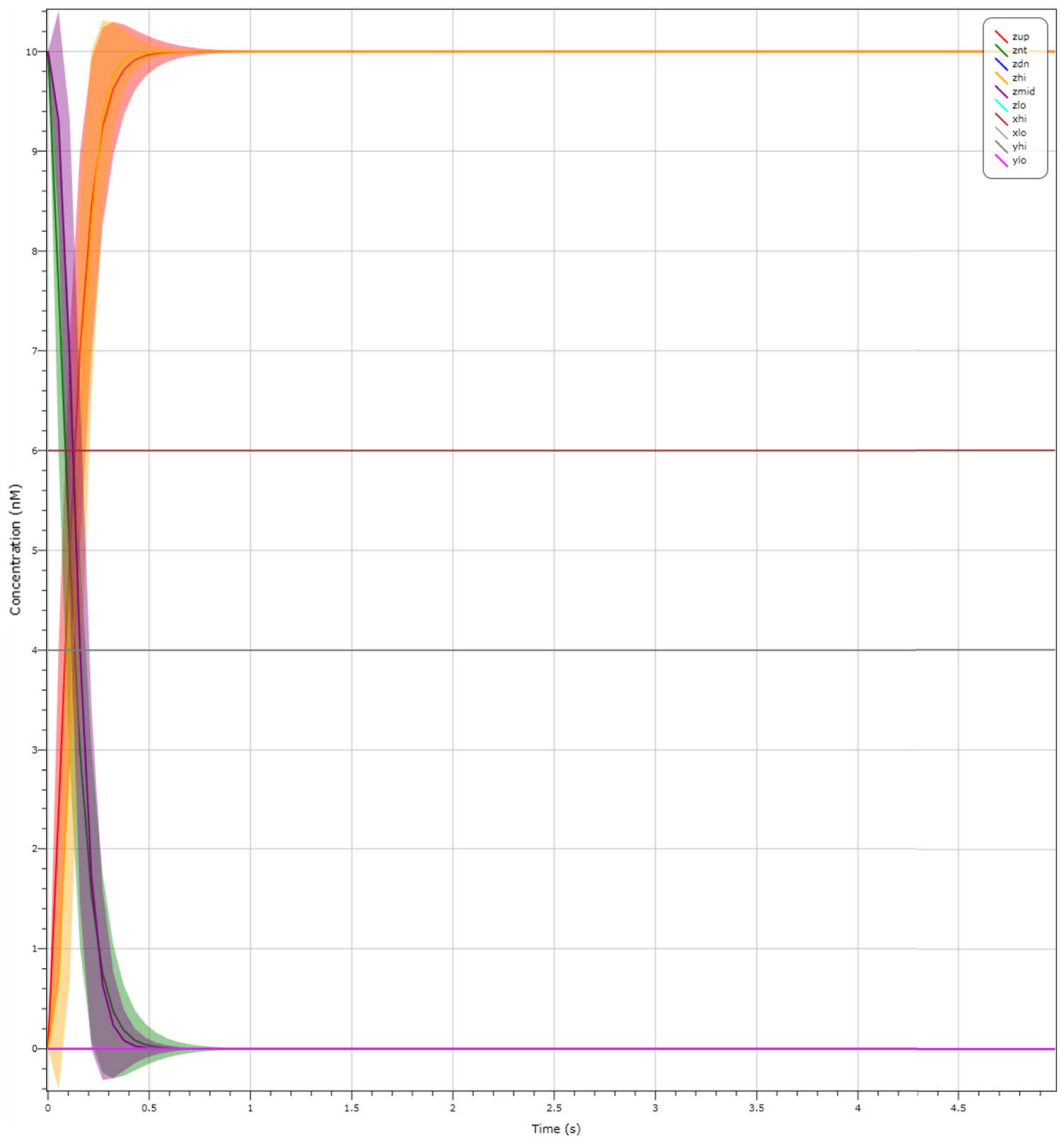
LNA simulation demonstrating two weak input signals, $X_{hi}$ and $Y_{hi}$ at 6 and 4 molecules respectively, boosted by the C-element with an output $Z_{hi}$ at 10 molecules

**Fig. 11:**
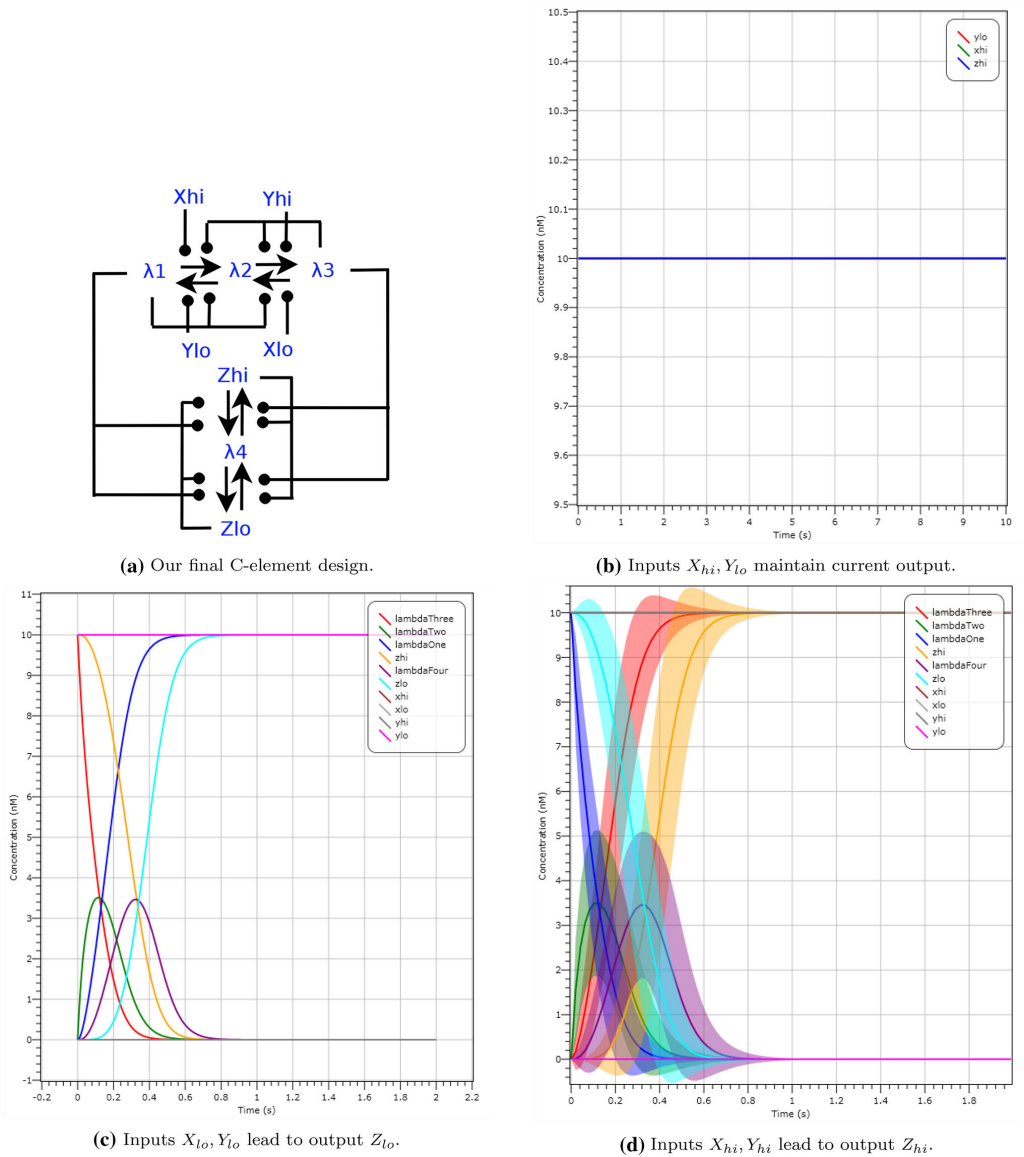
Simulation of the final dual-rail Muller C-element design on selected input configurations. **a** Dual-rail AM circuit which is our final C-element design. **b** Deterministic simulation with inputs $X_{lo}, Y_{hi}$ at 10 molecules and $Z_{hi}$ at 10 molecules, which does not exhibit any change over time. **c** Deterministic simulation resulting in $Z_{lo}$ (seen in light blue) based on the initial configuration $X_{lo}, Y_{lo}$ at 10 molecules and initial output $Z_{hi}$ at 10 molecules. **d** LNA simulation resulting in $Z_{hi}$ (seen in yellow) based on the initial configuration of input $X_{hi}, Y_{hi}$ at 10 molecules and initial output $Z_{lo}$ at 10 molecules. Note that some plots are overlayed but are either set to 0 or 10 molecules. (Color figure online)

An issue to address is the use of dual-rail systems in which our chemical output is precisely a value of 0 molecules or *K* molecules, where *K* is the largest number of molecules achievable by a population in the system. In reality, a species may not reach its maximum population and, due to variance, we may have a situation, as seen in Fig. 9, where one species has moderate probability of being higher than the output species we wish to present. Fortunately, we can use our approximate majority circuit to boost species. Figure 10 shows an example where species $X_{hi}$ and $Y_{hi}$ are weak, but the output is boosted by the C-element back to the maximum output of 10 molecules. The gates are also reusable: specifically, in Fig. 11d we can observe the inherent reusability of the C-element because the output reacts to the change in input.

### Latch design

**Fig. 12:**
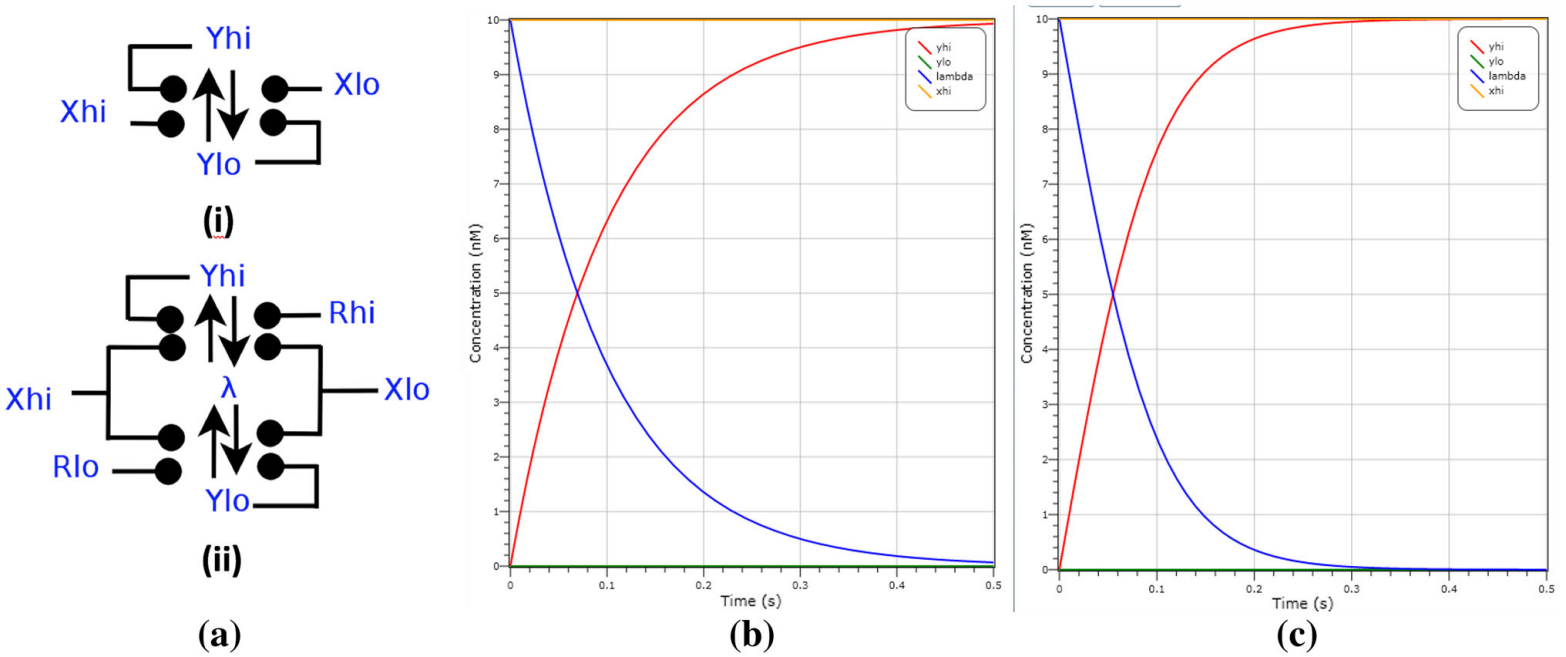
Two latch designs and their comparison. **a**(i) Simple latch with two feedback loops. **a**(ii) Latch with reset to a neutral state. **b** Deterministic plot of simple latch in **a**(i). **c** Deterministic plot of the latch in **a**(ii) which shows faster convergence

A latch is a device used in electronics to store a logical 0 or 1, which therefore needs to have at least two stable states that are cycled between. Latches are used in asynchronous computing both for storage and for synchronisation purposes. When an input of 1 is received a latch will ideally display an output of 1, and likewise for an input of 0. We present two latch designs in Fig. 12, each intended to interface in a specific way when used within a larger system. The first simple latch, shown in Fig. 12a(i), has input $X_{lo}, X_{hi}$ and output species $Y_{lo},Y_{hi}$, the intuition being that either $X_{lo}$ catalyses $Y_{hi}$ to $Y_{lo}$ or $X_{hi}$ catalyses $Y_{lo}$ to $Y_{hi}$. There are also two additional reactions that catalyse $Y_{lo}$ to itself and $Y_{hi}$ to itself, creating a feedback loop. These additional reactions ensure that, if there is a drop in the molecular concentrations of input species, the latch retains its state. For some larger systems we may need the output state of a latch to be neither $Y_{lo}$ nor $Y_{hi}$, to signify that no value is stored within the latch, known as a neutral state in electronics. A reset wire, to reset a latch to neutral state, is also commonly used in circuits. To this end, the latch in Fig.  12a(ii) has an input $R_{hi},R_{lo}$ used to reset the latch to a central state $\lambda$, as well as the standard inputs $X_{hi},X_{lo}$ and outputs $Y_{hi}, Y_{lo}$. The advantage of this central state, $\lambda$, is that the latch can be in a state where neither $Y_{hi}$ nor $Y_{lo}$ are present, which is useful if these reactions are catalytic to any other component, in which case they will not be triggered directly. A comparison of the behaviour of the two latches is displayed in Fig. 12b, c. With the same initial conditions $X_{hi}$ and output $Y_{lo}$ at 10 molecules, the latch in Fig. 12a(ii) produces an output $Y_{hi}$ at 10 molecules in 0.2 s. We contrast this with the latch in Fig. 12c, which outputs $Y_{hi}$ at 10 molecules in the slower time of 0.5 s.

### AM as a control flow element

An arbiter is used to decide an output signal based on which signal arrived first or if one signal is dominant over another. They are used in error correction where a signal may have degraded. Essentially, an arbiter computes the well known function $max(|X_1|,|X_2|)$ for two inputs. In terms of CRNs, this can be seen as either one species arriving before another or having higher molecular concentration. Since the AM circuit computes the $max(|X_1|,|X_2|)$ function, as one population is biased over

**Fig. 13:**
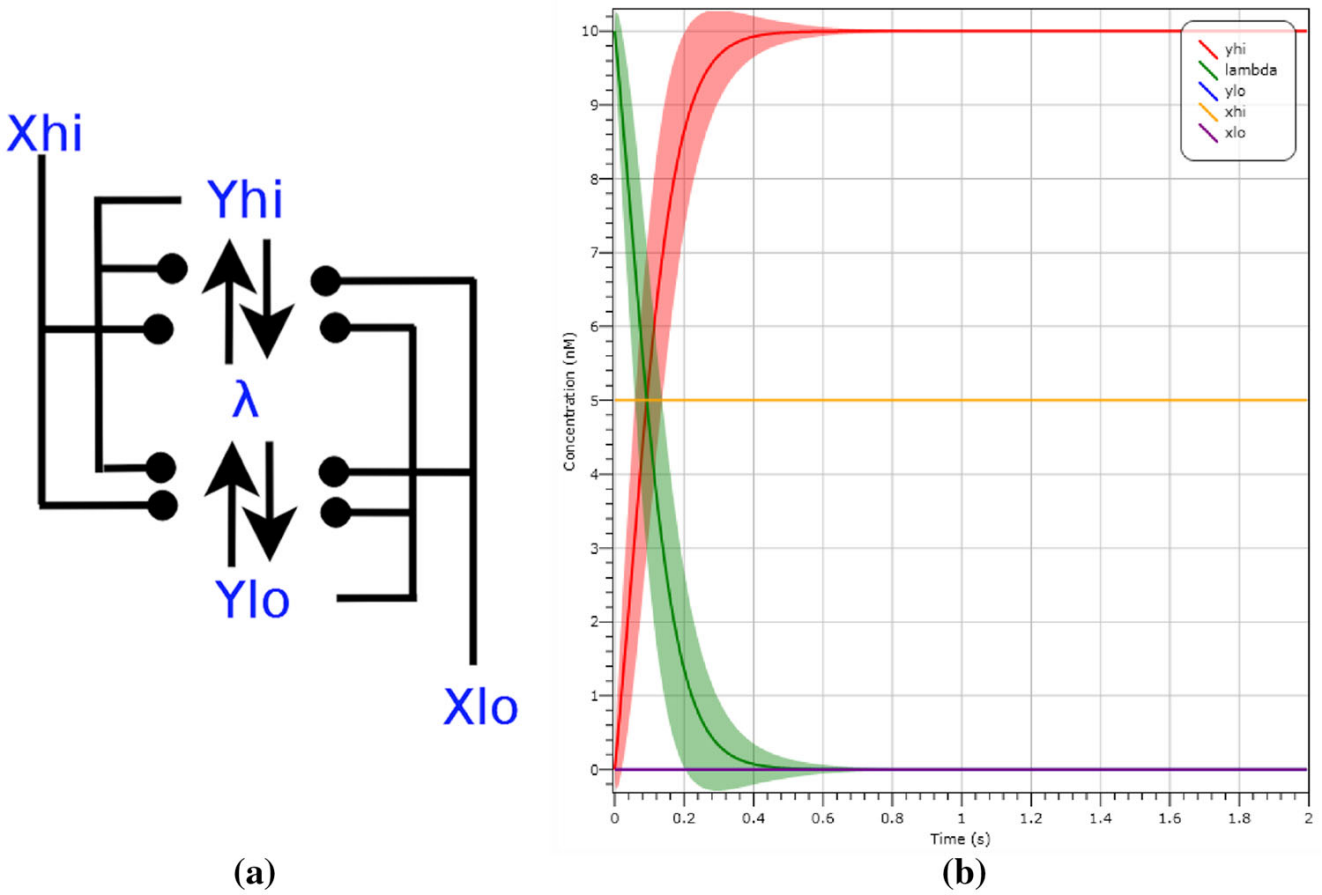
Arbiter circuit design and its simulation. **a** The arbiter CRN with inputs $X_{hi}$ and $X_{lo}$ and outputs $Y_{hi}$ and $Y_{lo}$. The output reflects the input with the higher concentration of molecules (or which ever species appeared first). **b** LNA simulation of the arbiter, demonstrated with an input of $X_{hi}$ at a concentration of 5 molecules and $X_{lo}$ with a concentration of 0 molecules. After 0.4 s we see $Y_{hi}$ (in red) at a concentration of 10 molecules, representing the majority input. (Color figure online)

another depending upon which has the majority, it therefore serves as an appropriate candidate for an arbiter. The proposed arbiter design, seen in Fig. 13a, is the same as our AM CRN presented in Fig. 8a, except that there are two inputs, $X_{hi}$ and $X_{lo}$, and two outputs, $Y_{hi}$ and $Y_{lo}$, instead of four inputs. This works as desired since the output $Y_{hi},Y_{lo}$ begins to be converted from $\lambda$ as soon as either of $X_{hi},X_{lo}$ arrives, therefore automatically biasing whichever species is present first. The ability for approximate majority to reach a consensus means that this circuit can deal with stochastic fluctuations in input. Although, within electronic circuits, an arbiter outputs which signal arrived first, we assume that this information is revealed through the promotion of an output species linked to an input species.

In Fig. 13b we demonstrate the operation of this arbiter by LNA simulation on inputs of $X_{hi}$ at a concentration of 5 molecules and $X_{lo}$ with a concentration of 0 molecules. After 0.4 s we see $Y_{hi}$ (in red) at a concentration of 10 molecules, representing the majority.

### Other control flow circuits

**Fig. 14:**
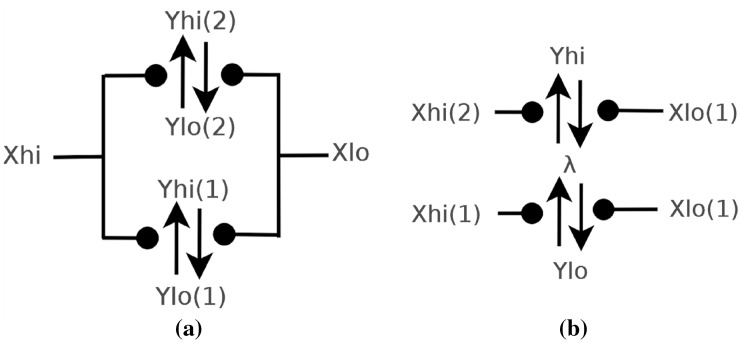
CRNs for control flow components. **a** The fork used to split a signal into two. **b** The join used to merge signals

Control flow is used to mediate or propagate the flow of information throughout the computing device. In digital circuitry forks and joins, both control flow elements, are naturally implemented using wires. Unfortunately, there is no natural fork or join within CRNs and consequently we present designs for them. The fork, shown Fig. 14a, is used to split signals. It has two input species $X_{hi}, X_{lo}$ and four output species, $Y_{hi}(1), Y_{hi}(2)$ to represent the splitting of $X_{hi}$ and $Y_{lo}(1), Y_{lo}(2)$ to represent the splitting of $X_{lo}$. The join, see Fig. 14b, joins two input signals to create one output signal. There are 4 inputs $X_{hi}(1), X_{hi}(2)$ with output $Y_{hi}$ to represent the merging of the two input signals and $X_{lo}(1), X_{lo}(2)$ to merge to an output $Y_{lo}$.

### Dual rail asynchronous logic gate designs

Although gate designs for Boolean operators have been proposed in CRNs (Soloveichik et al. 2008), we present dual-rail implementations of logic gates in line with other designs proposed within this paper. In contrast to the gates in Soloveichik et al. (2008), our gates account for all inputs $X_{hi},X_{lo}$, $Y_{hi}, Y_{lo}$ and outputs $Z_{hi}, Z_{lo}$. They are also reusable and respond to changes in input.

**Fig. 15:**
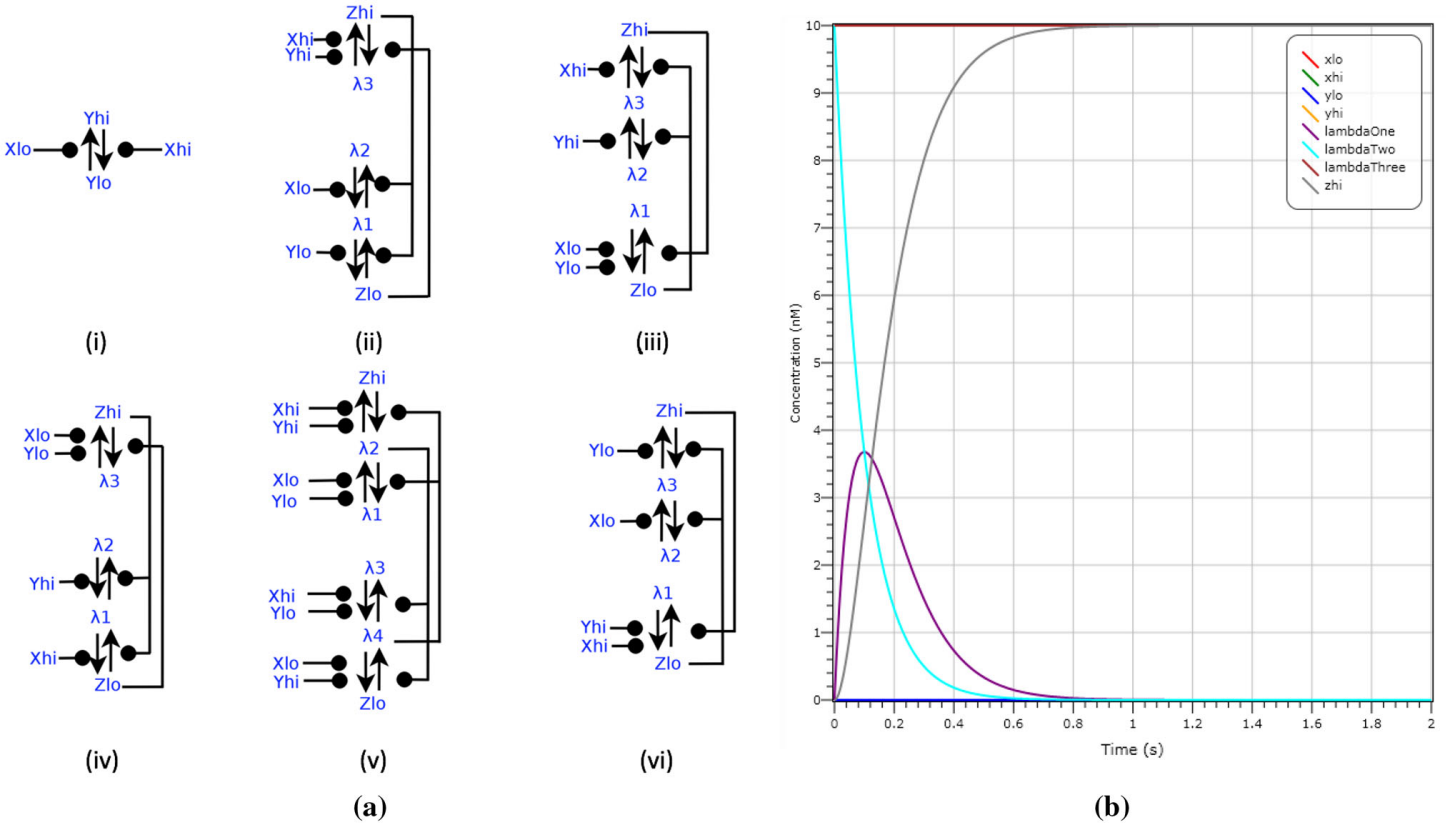
**a** Dual-rail logic gate designs: we present a NOT (i), OR (ii), AND (iii), NAND (iv), XOR (v) and NOR (vi) over inputs *X*, *Y* and output *Z*. **b** Deterministic simulation for AND on inputs $X_{hi},Y_{hi}$ at 10 molecules and initial output $Z_{lo}$, which is converted to $Z_{hi}$ after 0.8 s (seen in grey)

The simplest gate, NOT, in Fig. 15a(i), inverts the inputs $X_{hi}, X_{lo}$ to outputs $Y_{lo}, Y_{hi}$. The AND-gate, shown in Fig. 15a(iii), has initial concentrations of $\lambda _1,\lambda _2$ as well as input concentrations. With a presence of species $Y_{hi}$ we can catalyse $\lambda _2$ into the state $\lambda _1$, and with the species $X_{hi}$ we can catalyse $\lambda _1$ to $Z_{hi}$; thus both species are needed for the gate to output the signal $Z_{hi}$. The state $Z_{hi}$ catalyses a reaction between species $Z_{lo}$ and $\lambda _3$, therefore showing that only one output signal can be present at any time. Conversely, with either $X_{lo},Y_{lo}$ we can convert $\lambda _3$ to $Z_{lo}$, which in turn can convert $Z_{hi}$ back to $\lambda _2$ and $\lambda _2$ to $\lambda _1$. Using a similar trail of thought we can see how the other gates are devised, with OR (Fig. 15a(ii)) having initial concentrations of $\lambda _2, \lambda _3$, NOR (Fig. 15a(vi)) having initial concentrations of $\lambda _2, \lambda _3$ and NAND (Fig. 15a(iv)) having initial concentrations of $\lambda _1, \lambda _2$. We provide an example, showing a deterministic simulation of the AND gate, seen in Fig. 15b, in which inputs $X_{hi},Y_{hi}$ at 10 molecules and initial output $Z_{lo}$ is converted to $Z_{hi}$ after 0.8 s.

**Fig. 16:**
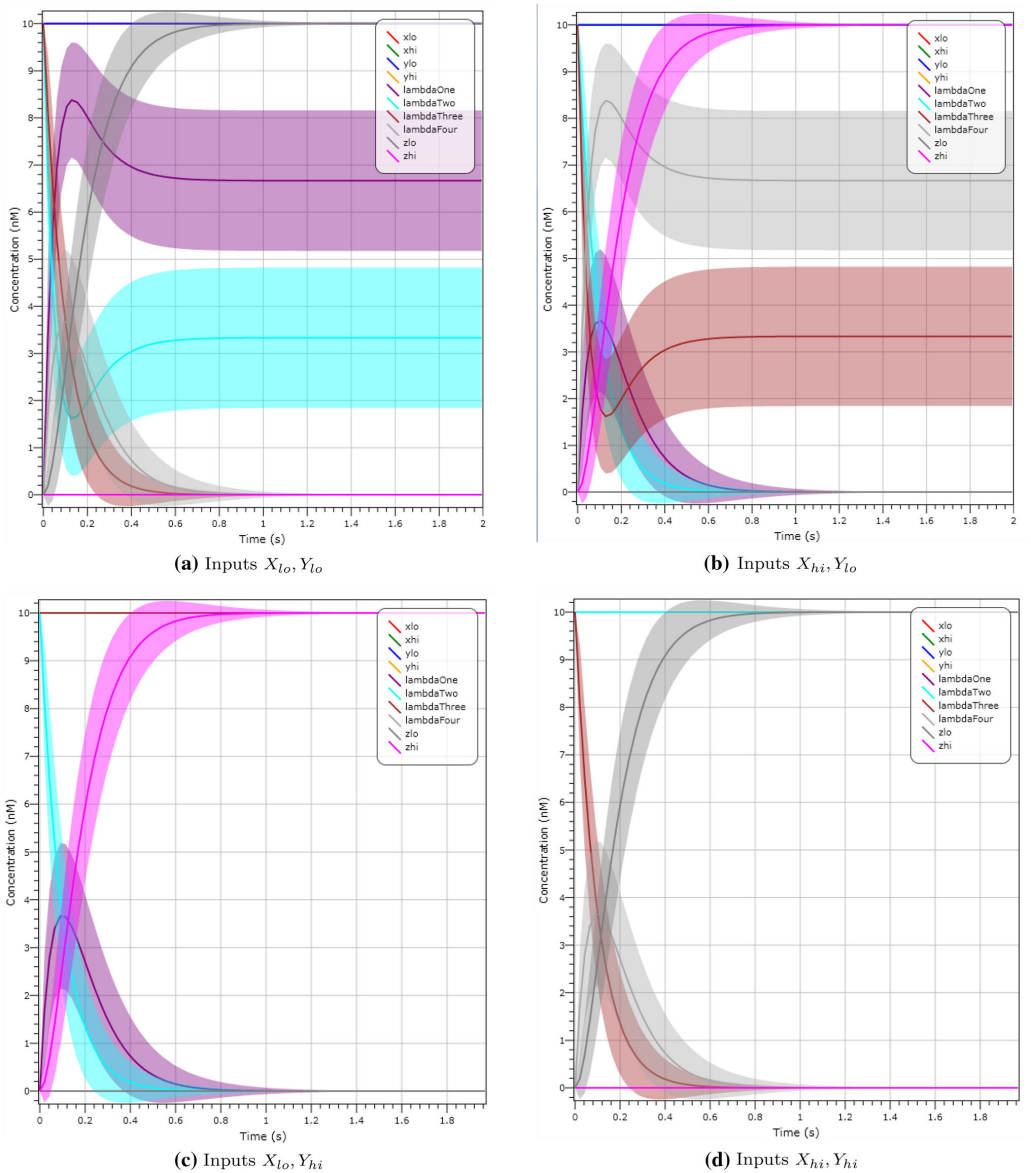
XOR gate validation demonstrated using LNA for all input combinations and $\lambda _1,\lambda _2,\lambda _3,\lambda _4$ having initial concentrations of 10 molecules. In **a** we demonstrate that XOR on an input configuration $X_{lo}, Y_{lo}$ at 10 molecules produces an output $Z_{lo}$ (seen in grey) at 10 molecules after 0.4 s. **d** A similar plot on input $X_{hi}, Y_{hi}$, which results in output $Z_{lo}$ (seen in grey). In **b**, **c** we show that both $X_{hi}, Y_{lo}$ and $X_{lo}, Y_{hi}$ demonstrate the correct output of $Z_{hi}$ (seen in pink). (Color figure online)

XOR is slightly different. XOR, traditionally a gate that requires a composition of many other logic gates, has to be constructed with all combinations of inputs considered. The XOR gate (Fig. 15a(v)) has initial concentrations of $\lambda _1,\lambda _2,\lambda _3,\lambda _4$. In Fig. 16 we show an example validation of the XOR gate for all four input configurations using LNA.

### Muller C-pipeline

We construct a CRN to emulate the behaviour of the C-pipeline outlined in Sect. 3.2. The pipeline is a mechanism to relay handshakes between components, for example latches to store data. We construct the pipeline by placing three of our C-element CRNs, shown Fig. 11a, in series. At each intermediate stage between the C-elements we add a fork. One path of the fork is negated and fed back into the previous C-element, and the other path is fed into the new C-element. The key interaction between the components of the C-pipeline is that no C-element can output a positive species or negative species without the previous displaying a positive or negative one.

The inputs to the C-pipeline CRN are $Req_{hi}, Req_{lo}, Acc_{hi}$ and $Acc_{lo}$. The only output is $C_{hi}, C_{lo}$, which is the output species of the third C-element. However, for the sake of clarity, we also include four other species $A_{hi}, A_{lo}$ corresponding to the output of the first C-element and $B_{hi}, B_{lo}$ corresponding to the second. On an input of $Req_{hi}$ at 10 molecules we would expect to see that $A_{hi}$, $B_{hi}$ and $C_{hi}$ are all at 10 molecules after a staggered amount of time. If we then changed the input to $Req_{lo}$ we would expect to see $A_{hi}$, $B_{hi}$, $C_{hi}$ diminish with $A_{lo}$, $B_{lo}$, $C_{lo}$, reaching 10 molecules to reflect the change in input. This is seen as a ‘wave’ through the pipeline propagating a high signal and then a low signal.

**Fig. 17:**
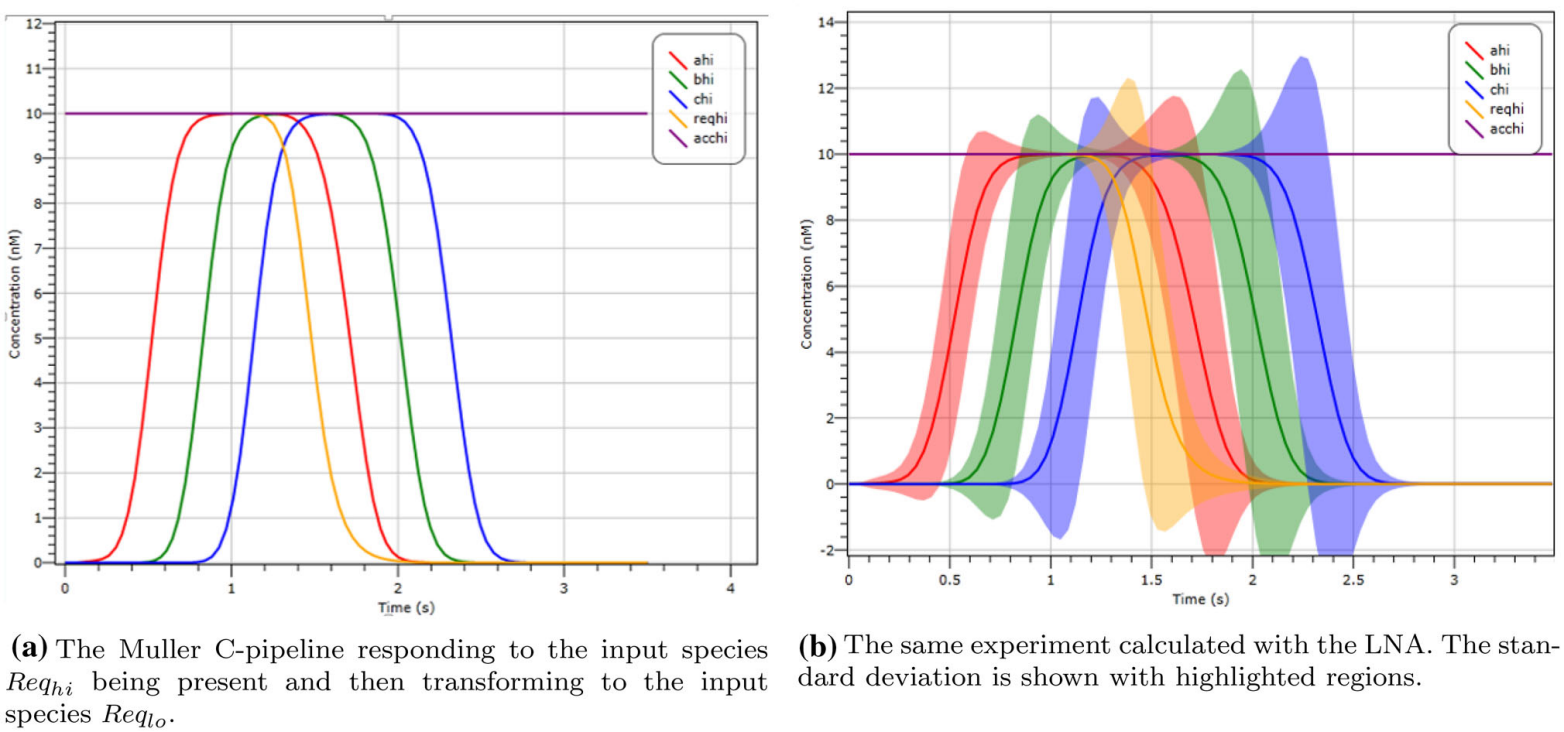
Validation of the Muller C-pipeline. The input request signal, encoded by the species $Req_{hi}$, is propagated to the end of the pipeline (represented by the species $A_{hi}, B_{hi}, C_{hi}$); we then set the request signal to low. The pipeline then responds by the presence of $A_{hi}, B_{hi}$ and $C_{hi}$ diminishing to zero. In **b** we show that the variance is low, even for low molecular counts

We design an experiment, see Fig. 17, where we initialise the pipeline with the input species $Req_{hi}$ at 10 molecules, and all C-elements are initialized with the intermediary species $\lambda$ at 10 molecules such that no C-element yet outputs a species. From both the deterministic and LNA simulations of this we can observe how the species $A_{hi}, B_{hi}, C_{hi}$ approach 10 molecules one after another, showing that indeed the value of $Req_{hi}$ is being propagated along the pipeline. In order to show that our pipeline design is continuously reactive, we convert all of the species $Req_{hi}$ to $Req_{lo}$ via the introduction of a reaction $Req_{hi} \rightarrow Req_{lo}$. This effect occurs at around 1 s and we can observe that the pipeline responds by reducing the species $A_{hi}, B_{hi}, C_{hi}$ to a molecular count of 0. We also observe (not shown on the simplified plot) that the species $A_{lo}, B_{lo}, C_{lo}$ reach 10 molecules at the same time as $A_{hi}, B_{hi}, C_{hi}$ reach 0 molecules (2 s). The LNA plot reveals that all output species are separated by a significant time difference such that no two species can be conflated.

**Fig. 18:**
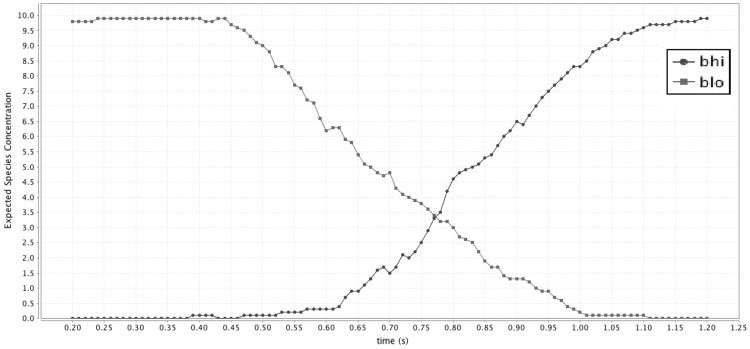
The expected concentration of species $B_{hi}$, $B_{lo}$ and intermediary $\lambda$ plotted over time for the C-pipeline under the stochastic semantics using reward structures within PRISM. Each data point is the expectation over 20 samples. We start at t = 0.2. With an initial condition of $Req_{hi}$, we can see (in blue) that species $B_{hi}$ is output from the second C-element at $t > 1.25$. (Color figure online)

In addition to simulation experiments which plot expected concentrations of species over time, we also check temporal properties concerning the interactions between species and components. Using the PRISM model checker we interrogate the CTMC models of the pipeline with specific queries. We give some important examples of such queries in Table 1, which are verified by PRISM as being true with very high probability by checking 20 paths against the property. “*Th*” refers to the required population threshold which can be set by the user. PRISM also has the ability to track and plot concentrations of species over specific time intervals and number of samples. For example, for the C-pipeline we may wish to focus specifically on species concentrations of the second C-element whilst ignoring the others. We can isolate the species in question starting at a time $> 0$ and simulating only the species of the second C-element. We demonstrate this property on the pipeline with initial input species $Req_{hi}$ at 10 molecules in Fig. 18.

**Table 1:** Temporal properties for the C-pipeline verified by the PRISM model checker. Each property was checked on 20 paths for the pipeline with inputs at 10 molecules

Property in English	Initial condition	PRISM query	Prob. of Success
“Probability that the first C-element always outputs a high signal before the second within 3 s”	$Req_{hi} > Th$	$P=?[(B_{hi} < Th) {\mathcal {U}}^{[0 , 3]}$ $(A_{hi} > Th)]$	0.97
“Probability that the C-element only changes when *both* inputs change within 10 s”	(1) $Req_{hi} Acc_{lo}$ (2) $Req_{lo} Acc_{hi}$ (3) $Req_{hi} Acc_{hi}$	$P=?[\text {true }{\mathcal {U}}^{[0,10]} Z_{hi} > Th ]$	0.96
“Probability that the species $A_{hi}$ reach their maximum population within 10 s”	$A_{hi} < Th$	$P=?[\text {true } {\mathcal {U}}^{[0,10]} A_{hi} >= Th]$	1
“Probability that request signal is propagated to the end of the pipeline within 10 s”	$Req_{hi} > Th$, $A_{hi}> Th$	$P=?[F^{[0,10]} C_{hi} > Th]$	1

### Queue

**Fig. 19:**
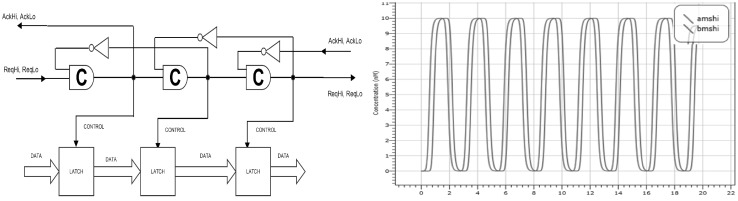
Deterministic simulation of the queue pipeline. We propagate a value of 1 through the queue. The species $Ams_{hi}, Bms_{hi}$ represent the outputs of the first and second latches. Note that through oscillatory patterns generated by the pipeline we can mimick properties of a synchronous system

We have also designed and validated a queue, the schematic for which is shown in Fig. 19, built by the addition of latches at each C-element block to the Muller pipeline. The queue is used in electronics to regulate and store the flow of information. The asynchronous queue uses the pipeline as a control mechanism to propagate signals between the latches. We use the latch with reset seen in Fig. 12 for this purpose. As the species $Req_{hi}$ is propagated along the pipeline, it sends a signal to the queue to read and store the value in the next latch along. Each latch represents some computation that could be completed within each time interval.

For the latches of our queue we have input species $Am_{hi}$ and $Am_{lo}$ and output species $Ams_{hi}, Bms_{hi},Cms_{hi}$ representing the output of each latch. Deterministic simulation the queue pipeline is shown in Fig. 19. In this experiment we propagate a 1 (represented as $Am_{hi}$ at 10 molecules) followed by 0 ($Am_{lo}$ at 10 molecules). We can observe the species $Ams_{hi}, Bms_{hi}$, which represent the outputs of the first and second latches, noting an oscillatory pattern of cycling between 1 and 0.

### Adder

**Fig. 20:**
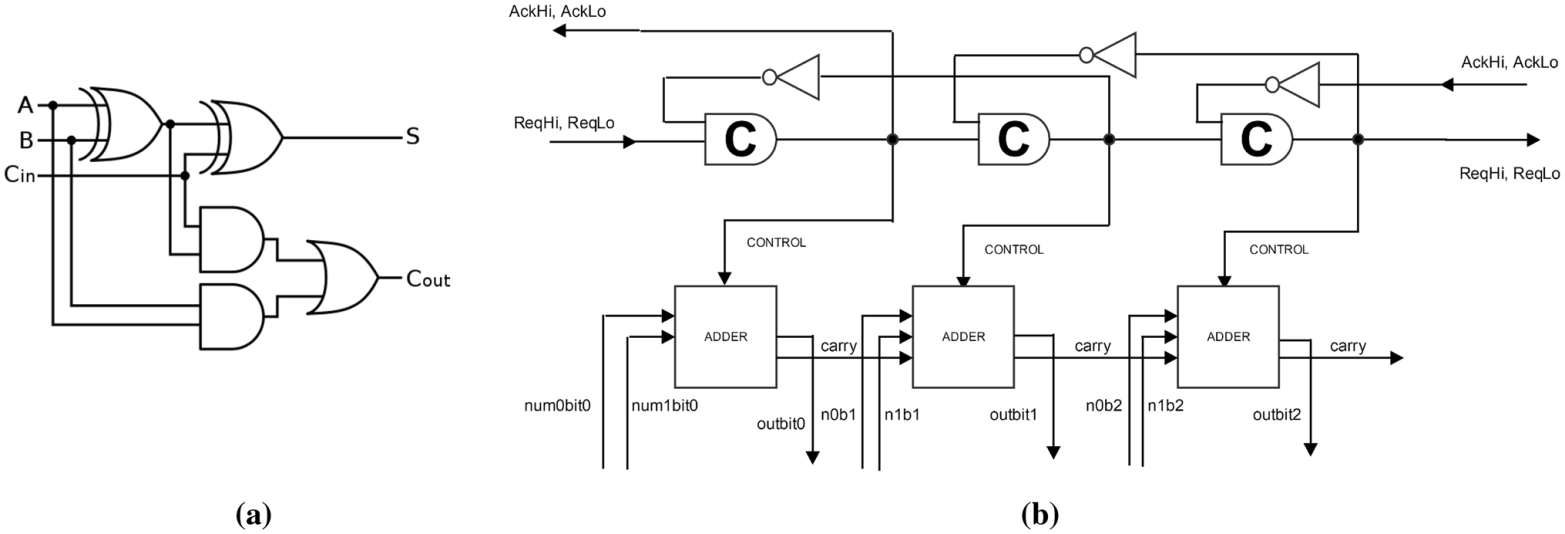
**a** Circuit diagram for a ripple-carry adder. **b** Three adders in series controlled by the C-pipeline. A carry-bit output from one adder is fed into the next as part of the input

We have also designed a three bit ripple-carry adder, which operates in a similar fashion to the queue but instead of latches we compose adders in series, seen in Fig. 20b. An adder, the circuit design for which is seen in Fig. 20a, is a composition of two XOR gates, two OR gates and an AND gate. The adder produces two outputs, the value of the summation and the carry. Within the ripple-carry adder, the carry output of each adder is fed into the next adder, which outputs the sum and a carry. In this way, with three adders, we can add three two-bit numbers together.

**Fig. 21:**
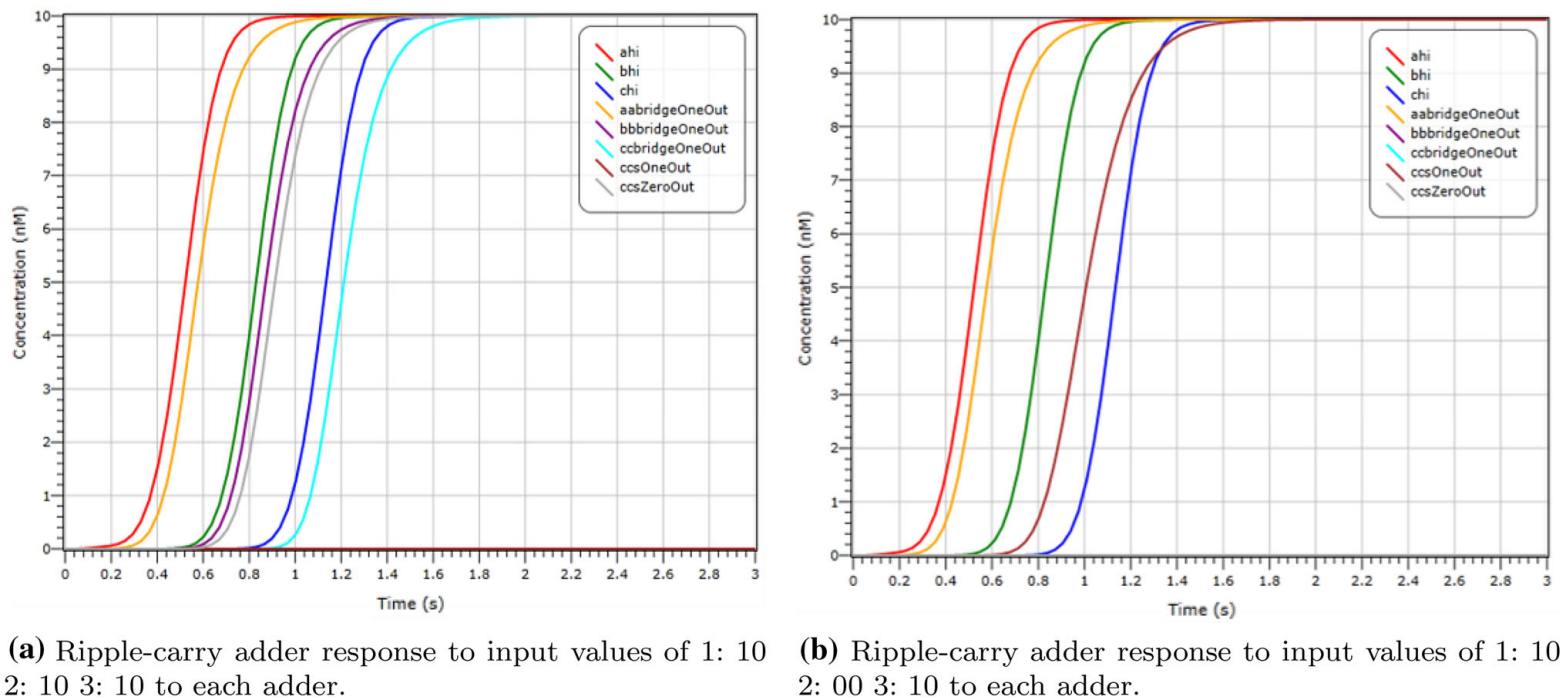
Deterministic simulation of the ripple-carry adder circuit responding to various inputs. We plot the output species from each section of the pipeline used to coordinate the output from each adder. The carry-bit output from each adder is represented by $A_{abridgeOneOut}, B_{bbridgeOneOut}$ and $C_{cbridgeOneOut}$. The output of the C-element ($A_{hi},B_{hi},C_{hi}$) arrives strictly before the output from the adder. The logical input for **a** is 1 and 0 for the first adder, 1 and 0 for the second adder, and 1 and 0 for the third adder. In **b** we show the computation on different inputs, namely 1 and 0 for the first adder, 0 and 0 for the second adder, and 1 and 0 for the third adder. The crossover in the concentrations of output species of the C-element and the logical output of 1 (resulting from inputs 0, 1 and carry of 1) in the third adder (plots $C_{hi}$ and $C_{csOneOut}$) indicates faster convergence but does not affect the results in further stages of the pipeline

Our ripple-carry CRN has four input species per adder representing the two inputs, and two output species representing the output. In Fig. 21 we show that the adder exhibits correct behaviour by producing the desired output species for a specific input, where each sum is calculated only in the next stage in the pipeline. If we view each C-element and adder as one stage in the pipeline, labelled A, B and C, then we can view the output species of each adder as a bridge to the next adder. The six output species, representing the carry-bit output, are denoted by $A_{abridgeOneOut}, B_{bbridgeOneOut}, C_{cbridgeOneOut}$ and $A_{abridgeZeroOut}, B_{bbridgeZeroOut}, C_{cbridgeZeroOut}$. In order to show correct operation the output of the adders (represented in this case by 10 molecules) should be interleaved with the control species of the C-pipeline ($A_{hi},B_{hi},C_{hi}$), allowing time for the carry species to catalyse with the input of the following adder.

Whilst a simulation provides the intuition behind the ripple-carry adder, using the PRISM model checker we can query the output of all three adders after 10 s to confirm if the correct output is present. We have four input species per adder, excluding the carry, which represent two numbers. We expect one species from each adder as output, representing the addition of two inputs, plus the carry. The third adder relies on the previous adder’s carry being correct. We therefore only need to look at the desired output of each adder plus the carry of the final adder. We summarise this with the following example PRISM property:

$$\begin{aligned}&P =? [ \text { true } U <= 10 ( (A_{abridgeOneOut}> Th) \text { and }\\&(B_{bbridgeZeroOut}> Th) \text { and } ( C_{cbridgeOneOut}> Th) \text { and }\\&(C_{cbridgeCarryOneOut} > Th) ) ] \end{aligned}$$

With this example we can only satisfy these three outputs and the carry, by seeing each of their molecular concentrations rise above the threshold *Th*, based on a specific input configuration. This particular input are the species representing 0 and 1 for the first adder (for example $A_{aOneZeroIn}$ and $A_{aTwoOneIn}$), the species representing inputs 1,1 for the second adder and 1,1 for the third adder. $0 + 1$ in the first adder should give us an outcome of 1 carry 0 and so satisfies $A_{abridgeOneOut} > Th$. $1 + 1$ plus the 0 carry from the first adder gives an output of 0 carry 1, and so satisfies $B_{bbridgeZeroOut} > Th$. An input of 1 + 1 plus 1 carry from the second adder means that our output should be 1 as well as the final carry should be 1, represented by $C_{cbridgeOneOut} > Th$ and $C_{cbridgeCarryOneOut} > Th$. Our adder satisfies this property based upon the inputs given and therefore shows correct operation for an adder.

## Experimentation

All designs^3^ presented in this paper have been validated using both Microsoft’s Visual GEC tool (Pedersen and Phillips 2009) and the PRISM model checker (Kwiatkowska et al. 2011), both for the deterministic and stochastic semantics of the CRNs. Visual GEC provides a programming language, LBS, for designing and simulating any given CRN. We systematically tested each component in isolation by simulating its behaviour against all input and output configurations. Next, we examined how a component might behave in a larger system, where it will be exposed to a change in input. To this end, we introduced new reactions to emulate a signal change. For instance, if we wished to change a carrier signal from high to low, we would introduce an additional reaction $X_{hi} \overset{k}{\rightarrow } X_{lo}$, which converts all of the signal $X_{hi}$ into a signal $X_{lo}$ while the component is operating.

Since deterministic semantics is not accurate for low molecular populations, we additionally explored stochastic semantics. Visual GEC exports models to the probabilistic model checker PRISM, which then enables verification of the induced continuous-time Markov chain against temporal logic properties. This allows one to check that the circuits ensure the correct temporal ordering of the events, for example, for the Muller pipeline seen in Fig. 6, that the species in the first stage of the pipeline is present before the species in the second, i.e. with probability 1, and that the signal is eventually propagated to the end of the pipeline. PRISM implements numerical solution of the CME, which is exponential in the initial number of molecules and hence not scalable, and analysis based on stochastic simulation, which is time consuming. We thus additionally used an experimental implementation of the LNA within Visual GEC, based on Cardelli et al. (2015). As well as being capable of checking temporal logic properties (Cardelli et al. 2015; Bortolussi et al. 2016), the LNA can plot the species concentration over time together with standard deviation, and is fast and reasonably accurate even for low molecule counts. Moreover, compared to the deterministic semantics, LNA provides important information about stochasticity that may affect the robustness of the circuits, and which can be explored further with CME, stochastic simulation, or verifying that the circuit converges with probability 1 to a single value.

## Discussion

When modelling asynchronous circuits as a chemical system, the wires are chemical species from the output set of one gate component to the input set of another. We cannot bound the time for a gate to transform an input species to an output species. This excludes the class of self-timed circuits. Under deterministic semantics, we could guarantee an isochronous fork since two chemical species, either high or low, could theoretically reach the threshold *M* at precisely the same time given equal rates and initial concentrations, and therefore under deterministic semantics we have a Turing-complete method of computation. We cannot guarantee this under stochastic semantics (Cook et al. 2009). This is because there is a non-zero probability that one species could reach *M* before the other. We thus conclude that our circuits, at worst, can be classified as speed independent. We can calculate approximately the delay on wires based upon rates and concentrations for each semantics.

Direct chemical implementations of CRNs have been theorised and realised, but involve complicated reaction mechanisms (Shin 2011). The most common substrate for chemical kinetics is DNA strand displacement (DSD), which involves the displacement of DNA strands in solution. These strands are labelled with the chemical species and, once the reaction has taken place, an outputting strand represents an output from the CRN that the strand displacement system is trying to emulate. DNA strand displacement has been shown to be a universal substrate for chemical kinetics, specifically for bi-molecular reactions used here (Soloveichik et al. 2010). Most importantly, the AM circuit seen in Fig. 8b has been implemented as a strand displacement device (Lakin et al. 2012). However, a potential difficulty with this approach is scalability: as the number of components increases, the number of chemical species representing them also increases. Large numbers of chemical species result in large numbers of DSD complexes in solution, and consequently crosstalk needs to be accounted for. Fortunately, a recent experiment with the implementation of a square-root circuit in solution provided a new experimental ceiling on the number of species that can be used (Qian and Winfree 2011).

Another challenge is to provide formal verification of the correctness of the designs. We remark that, although we have validated the behaviour of the components for all possible input configurations and verified that correct outputs are produced and in the correct order using simulation and simulation-based probabilistic model checking with PRISM, this does not amount to full verification. Asynchronous diagrams are represented using a variety of notations, see Fig. 5, and correspond to certain classes of (safe) Petri nets (Myers 2004) known as Signal Transition Graphs (STGs), representing the rise and fall of signals. Our designs are systems containing many molecules which exhibit stochastic behaviour. Firstly, one would need to show that our CRN designs meet the specification given as a Petri net, which would involve relating the two formally via a refinement relation, where one needs to relate structures with many molecules of a given a species to structures with at most one. This presents us with two major challenges: scalability and stochasticity. Scalability can be addressed using compositional verification, which has been developed for (non-probabilistic) process algebraic specifications of asynchronous circuits (Wang and Kwiatkowska 2007) (equivalent to STGs) and it would be interesting to see if they can be applied in this setting. However, no probabilistic extension of this approach is known. Another possibility is to capture stochasticity by employing stochastic Petri nets to model the designs as done for molecular walkers in Barbot and Kwiatkowska (2015), and then perform temporal logic verification using the tool Cosmos. Cosmos relies on an implementation of statistical model checking that exploits parallelism of the Petri net specifications and achieves greater scalability than PRISM.

## Conclusion

We have proposed a novel design for an asynchronous computing device based on Chemical Reaction Networks. CRNs are inherently asynchronous, and thus particularly well suited to this computational paradigm. Our designs are based on a simple, bi-molecular reaction motif inspired by Approximate Majority (Angluin et al. 2008; Cardelli and Csikász-Nagy 2012), employ catalytic reactions and assume well-mixed solution and constant, uniform rates. Moreover, they do not rely on the universal clock which is difficult to realise. Since an arbitrary CRN can be physically realised using DNA strand displacement (Soloveichik et al. 2010), as recently demonstrated experimentally in Chen et al. (2013), the proposed designs are in principle implementable, and we have confirmed this in theory by modelling them in the two-domain setting (Cardelli 2010) using Visual DSD (Phillips and Cardelli 2009; Lakin et al. 2011). Our designs are the first feasible implementation of an asynchronous computing device in chemical kinetics and are relevant for a multitude of applications in nanotechnology and synthetic biology. As future work we would like to investigate alternative experimental settings.

## Footnotes

1
http://lepton.research.microsoft.com/webgec/ (Pedersen and Phillips 2009), both for the deterministic and stochastic semantics.

2
www.prismmodelchecker.org.

3 Available from https://github.com/max1s/CRNcode.
